# The activity of CobB1 protein deacetylase contributes to nucleoid compaction in *Streptomyces venezuelae* spores by increasing HupS affinity for DNA

**DOI:** 10.1093/nar/gkae418

**Published:** 2024-05-23

**Authors:** Julia Duława-Kobeluszczyk, Agnieszka Strzałka, Michał Tracz, Magdalena Bartyńska, Katarzyna Pawlikiewicz, Tomasz Łebkowski, Sara Wróbel, Justyna Szymczak, Anna Zarek, Tomasz Małecki, Dagmara Jakimowicz, Marcin J Szafran

**Affiliations:** Faculty of Biotechnology, University of Wrocław, 50-383 Wrocław, Poland; Faculty of Biotechnology, University of Wrocław, 50-383 Wrocław, Poland; Faculty of Biotechnology, University of Wrocław, 50-383 Wrocław, Poland; Faculty of Biotechnology, University of Wrocław, 50-383 Wrocław, Poland; Faculty of Biotechnology, University of Wrocław, 50-383 Wrocław, Poland; Faculty of Biotechnology, University of Wrocław, 50-383 Wrocław, Poland; Faculty of Biotechnology, University of Wrocław, 50-383 Wrocław, Poland; Faculty of Biotechnology, University of Wrocław, 50-383 Wrocław, Poland; Faculty of Biotechnology, University of Wrocław, 50-383 Wrocław, Poland; Faculty of Biotechnology, University of Wrocław, 50-383 Wrocław, Poland; Faculty of Biotechnology, University of Wrocław, 50-383 Wrocław, Poland; Faculty of Biotechnology, University of Wrocław, 50-383 Wrocław, Poland

## Abstract

*Streptomyces* are soil bacteria with complex life cycle. During sporulation *Streptomyces* linear chromosomes become highly compacted so that the genetic material fits within limited spore volume. The key players in this process are nucleoid-associated proteins (NAPs). Among them, HU (heat unstable) proteins are the most abundant NAPs in the cell and the most conserved in bacteria. HupS, one of the two HU homologues encoded by the *Streptomyces* genome, is the best-studied spore-associated NAP. In contrast to other HU homologues, HupS contains a long, C-terminal domain that is extremely rich in lysine repeats (LR domain) similar to eukaryotic histone H2B and mycobacterial HupB protein. Here, we have investigated, whether lysine residues in HupS are posttranslationally modified by reversible lysine acetylation. We have confirmed that *Streptomyces venezuelae* HupS is acetylated *in vivo*. We showed that HupS binding to DNA *in vitro* is controlled by the acetylation. Moreover, we identified that CobB1, one of two Sir2 homologues in *Streptomyces*, controls HupS acetylation levels *in vivo*. We demonstrate that the elimination of CobB1 increases HupS mobility, reduces chromosome compaction in spores, and affects spores maturation. Thus, our studies indicate that HupS acetylation affects its function by diminishing DNA binding and disturbing chromosome organization.

## Graphical Abstract

**Graphical Abstract:**
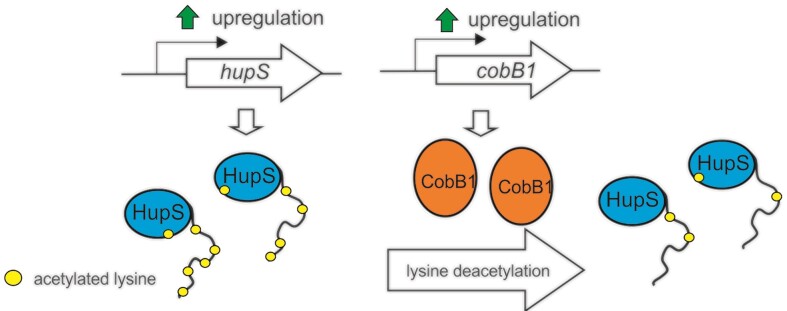

## Introduction

All living organisms face the requirement of compacting their genetic material inside the cell or nucleus while concomitantly providing access for DNA transactions such as chromosome replication and partitioning, transcription, or DNA repair. Bacteria condense their chromosomes approximately 1000-fold into a ‘nucleoid’ structure suspended within the cytoplasm (1–4). The levels of DNA compaction become even higher in sporulating bacteria, whose chromosomes are up to 3-fold more compacted within spores than during vegetative growth (5). Among several factors contributing to chromosome packaging, nucleoid-associated proteins (NAPs) are one of the key players (6–8). NAPs are a highly heterogeneous group of small, basic proteins that bind DNA without (or with very low) sequence preferences and promote DNA wrapping, bending or bridging (4,8–10). Due to functional similarities, NAPs are often referred to as histone-like proteins, although their homology with eukaryotic histones is low. The most conserved and abundant NAPs in bacteria are HU (*heat unstable*) homologues (11,12). In general, the cellular repertoire of NAPs often differs among bacterial species. Moreover, the levels of NAPs can change notably in response to altered growth conditions or environmental signals, making NAPs the major remodellers of bacterial nucleoids (13).

*Streptomyces*, as soil-dwelling bacteria, are particularly exposed to environmental stressors. In response to stress conditions, *Streptomyces* produce secondary metabolites, many of which have valuable antibacterial and antifungal activities. Environmental stress, particularly nutrient limitation, also induces sporulation, which is the final stage of their fungal-like development. *Streptomyces* development starts with spore germination, followed by the growth of branched, multigenomic vegetative hyphae. Sporulation is initiated by the formation of unbranched aerial (sporogenic) hyphae. The life cycle ends with the transformation of multigenomic aerial hyphae into long chains of unigenomic exospores (14). *Streptomyces* differentiation is tightly controlled by the *bld* and *whi* regulatory cascades that synchronize the particular stages of hyphal growth with chromosome segregation and compaction (5,15–17). The structure of *Streptomyces* linear chromosomes changes dramatically during their complex life cycle, from uncondensed in vegetative hyphae to highly compacted within spores (18). The gradual rearrangements of their chromosome are executed by the concerted action of NAPs and condensins. Although extensively studied, the role of NAPs during *Streptomyces* sporulation is complex and still not fully understood. Most of the NAPs studied thus far (i.e. DpsA, DdbA, sIHF, HupS) enhance chromosome compaction in spores, and their removal leads to an increase in the nucleoid volume; however, the elimination of others (DpsB, DpsC) shows the opposite effect (5).

Among several *Streptomyces* NAPs that are activated during sporogenic development, the HupS protein is the best studied. HupS is one of two (apart from the HupA protein) HU homologues encoded in *Streptomyces* genomes (19). The HupS protein is composed of two domains. Its N-terminal domain shows homology to the *Streptomyces* HupA protein and *Escherichia coli* HU homologue. On the other hand, the C-terminal domain of HupS resembles the eukaryotic histones or mycobacterial HupB homologue (also referred to as Hlp), as it contains multiple lysine repeats (LR domain) (20,21). Although both HupS and HupB proteins do not show sequence specificity in DNA binding, they exhibit nonuniform distribution along the chromosome. Whereas in *Streptomyces venezuelae* HupS binding increases towards the chromosomal termini (*ter*), the enrichment of *Mycobacterium smegmatis* HupB binding sites within the circular mycobacterial chromosome shows an opposite pattern, with elevated binding in proximity to the *oriC* region (18,22). The deletion of the *hupS* gene in *Streptomyces coelicolor* and *S. venezuelae* increased nucleoid volume in spores (18,19). In *S. coelicolor*, HupS elimination was also shown to lower spore resistance to heat and UV radiation (19). In mycobacteria, deletion of the *hupB* gene also increased sensitivity to environmental stresses, including cold shock, UV radiation, exposure to isoniazid, and host persistence (23–25). Interestingly, the C-terminal truncation of HupB resulted in abolished DNA affinity and led to nucleoid decompaction in *M. smegmatis*, thus revealing a similar phenotype to the *hupB* null mutant (22,26). The identification of LR domains only in DNA binding proteins reinforces their significance for protein−DNA interactions (27). Markedly, the LR domain of HupB has been shown recently to be a target for posttranslational modifications (PTMs), including reversible multiple lysine methylation and acetylation (28,29). *M. tuberculosis* strains lacking acetyl- and methyltransferases that modify the LR domain of HupB showed increased susceptibility to antibiotics and lowered host persistence, characteristic of *hupB-*deficient strains (29–31).

Reversible lysine acetylation (RLA) allows immediate up- or downregulation of protein activities in response to internal or environmental stimuli, bypassing energy-consuming *de novo* protein synthesis or degradation (32). Ubiquitous in bacteria, lysine acetylation can occur spontaneously in the cytoplasm or can be catalysed by acetyltransferases (Pat), which are homologues of the yeast Gcn5 histone N-acetyltransferase (GNAT) (33). Both lysine acetylation pathways depend on the intracellular availability of acetyl phosphate (AcP) or acetyl-coenzyme A (acetyl-CoA). AcP and acetyl-CoA are two high-energy metabolites serving as cytoplasmic donors of acetyl groups, directly linking primary metabolism with the global regulation of protein activities. Lysine acetylation can be reversed only enzymatically, requiring the activity of protein deacetylases. Bacterial deacetylates belong to two groups, depending on the NAD^+^ requirements. CobB deacetylases (also referred to as SrtA) are homologues of the yeast Sir2 protein and require NAD^+^ as a cofactor. On the other hand, AcuC homologues coordinate Zn(II) to catalyse the transacetylation reaction in an NAD^+^-independent manner(32,34,35).

In *S. coelicolor*, two Sir2 homologues have been identified: CobB1 (SCO0452) and CobB2 (SCO6464). Whereas CobB1 has lysine deacetylase activity *in vitro*, CobB2 has been reported recently to have a preference for succinate groups attached to the side chain of a lysine residue and was suggested to play a role as a cellular protein desuccinylase with poor deacetylation activity (36,37). Interestingly, the identification of 601 and 667 acetylated proteins in *S. coelicolor* and *Streptomyces roseosporus*, respectively, indicates that reversible lysine acetylation may be a widespread mechanism controlling protein activity in these bacteria and suggests the presence of many potential protein substrates for CobB1 protein deacetylase (37,38).

The presence of the LR domain, which is extremely rich in lysine residues, in HupS suggests that its posttranscriptional regulation is similar to that detected for mycobacterial HupB. Therefore, we aimed to test whether HupS is modified by lysine acetylation and whether such a modification could affect the interaction of HupS with DNA. Having confirmed that HupS is the target of acetylation, by combining *in vivo* and *in vitro* studies, we showed that increased HupS acetylation lowers its DNA affinity, leading to nucleoid decompaction in *S. venezuelae* spores. Moreover, we confirmed that lysine-acetylated HupS is a substrate for the CobB1 protein deacetylase, whose levels increase during *S. venezuelae* sporogenic differentiation. Thus, we postulate that CobB1 serves as a regulator of HupS-DNA binding in *S. venezuelae*, promoting HupS-dependent chromosome organization during sporulation. In summary, our studies expand the scope of knowledge on the role of PTMs in the regulation of chromosome organization in sporulating bacteria.

## Materials and methods

### Bacterial strains and plasmids

The *Escherichia coli* and *S. venezuelae* strains used in this study are listed in the Supplementary Materials and Methods (Supplementary Tables S1 and S2). The oligonucleotides and plasmids are listed in the Supplementary Materials and Methods (Supplementary Tables S3 and S4). The culture conditions, antibiotic concentrations, and transformation and conjugation methods followed the general procedures for *E. coli* (39) and *Streptomyces* (40). All DNA manipulations were performed according to standard procedures (39) or the manufacturer's protocols. The DNA-modifying enzymes, restriction enzymes, and DNA polymerases were purchased from New England Biolabs (US) or Thermo Fisher Scientific (USA). The oligonucleotides were synthesized by Genomed S.A. (Poland) or Sigma−Aldrich (US). The details of plasmid and strain constructions as well as the protein purification protocols are described in the Supplementary Materials and Methods.

### Growth analyses

*S. venezuelae* strains were cultivated on MYM (solid or liquid), SFM, or minimal (MM) medium not supplemented with mannitol. The media and antibiotics were purchased from Carl Roth (Germany), Gibco (USA), Becton Dickinson (USA), and A&A Biotechnology (Poland). To obtain the *S. venezuelae* growth curves, a Bioscreen C instrument (Growth Curves, USA) was used. *S. venezuelae* cell cultures were set up in sterile honey-comb microplates (Growth Curves, US) in 300 μl liquid MYM medium inoculated with 0.0001 U of spores (1 U is an optical density (OD_600_) of the spore stock solution equal to 1 when measured in miliQ water). Each strain was cultured in three independent experimental repeats for 48 hours at 30°C under the ‘medium’ speed and ‘normal’ shaking amplitude settings. The growth of *S. venezuelae* was monitored by optical density (OD_600_) measurements in 20-min intervals. The data were collected using BioScreener 3.0.0 software and the growth curves were plotted using Microsoft Excel.

For *S. venezuelae* growth and development analyses on solid plates, the spore suspensions in 10% glycerol were diluted with sterile water to reach a spore concentration of 0.001 U/μl. Then, 5 μl of spore dilution was plated onto MYM, SFM or MM, and incubated at 30°C for up to 72 h (growth analysis) or 120 h (spore pigmentation).

To assess the spore germination efficiency, the frozen spore aliquots were thawed on ice, the spores were counted under microscope using Thom's chamber, and subsequently diluted (10^6^–10^8^ times) with chilled 10% glycerol. Next, 50 μl of each spore dilution was spread on MYM plate and incubated at 30°C for 48 hours. The experiment was performed as 3–4 replicates. The obtained colonies were counted and the average CFU (*colony forming units*) was calculated. The germination efficiency (%) is the average number of CFU shown as a fraction of the total number of spores streaked on the plate.

### Western blotting

For Western blotting analyses, *S. venezuelae* cultures were set up as follows: 5 ml of liquid MYM was inoculated with 0.05 U of spores, and the cultures were incubated for 8–25 h at 30°C with shaking (180 rpm). Next, a 2 ml sample of the culture was collected by centrifugation (5000 g, 5 min, 4°C), washed twice with phosphate-buffered saline (PBS buffer (38)), resuspended in 300 μl of chilled PBS buffer supplemented with Pierce™ Protease Inhibitor Tablets (Thermo Fisher Scientific, USA), and disrupted by sonication. The cell lysate was then clarified by centrifugation (12 000 g, 5 min, 4°C), and the supernatant was transferred to a fresh tube. The total protein concentration was quantified using the ROTI^®^Quant Universal kit (Carl Roth, Germany). An appropriate volume containing 10–20 μg of cell lysate proteins was mixed with 6x SB buffer (375 mM Tris–HCl pH 6.8, 12% SDS, 0.06% bromophenol blue, 600 mM DTT, 60% glycerol), denatured at 95°C for 10 min, and resolved by standard Laemmli acrylamide gel electrophoresis (SDS-PAGE). After electrophoresis, proteins were stained with InstantBlue^®^ Coomassie Protein Stain (Abcam, UK) (CBB, loading control) or transferred to a nitrocellulose membrane (Amersham, UK) and blocked with 2% skim milk (SM Gostyń, Poland) in Tris-buffered saline (TBS buffer) supplemented with 0.05% Tween-20 (TBST buffer) (39). The blocked membrane was subsequently incubated with primary rabbit polyclonal anti-AcK antibody (Cell Signaling Technology, US) for acetyllysine detection, mouse monoclonal anti-HisTag antibody (Invitrogen, US) for HisTag detection, mouse monoclonal anti-FLAG antibody (Sigma−Aldrich, US) for FLAG-Tag detection, or rabbit anti-TopA serum (41) for TopA detection. Primary antibodies were diluted 1:500 (anti-AcK, anti-HisTag), 1:1000 (anti-FLAG), or 1:5000 (anti-TopA) in TBST buffer, and the membrane was incubated for 1 hour at room temperature followed by washing the membrane with 5 ml of fresh TBST buffer for 5–10 min at room temperature (repeated 3 times). Next, the membrane was incubated with secondary polyclonal antibodies: anti-mouse IgG conjugated with horseradish peroxidase (HRP, Invitrogen, US), anti-rabbit IgG conjugated with horseradish peroxidase (Invitrogen, US) or alkaline phosphatase (AP, Sigma−Aldrich, USA). The secondary antibodies were diluted 1:5000 (HRP-conjugated) or 1:10 000 (AP-conjugated) in TBST buffer. Next, the membrane was incubated and washed as described earlier. For HRP signal detection, the membrane was washed briefly with 5 ml of SuperSignal West Pico PLUS Chemiluminescent Substrate solution (Thermo Fisher Scientific, USA), and the chemiluminescence signal was quantified using ChemiDoc XRS+ (Bio-Rad, USA). For AP activity detection, the membrane was incubated for 5–10 min with AP buffer (100 mM Tris–HCl pH 9.5, 100 mM NaCl, 5 mM MgCl_2_, 0.05% Tween 20) supplemented with 0.175 mg/ml 5-bromo-4-chloro-3-indolyl-phosphate (BCIP) and 0.225 mg/ml 4-nitro blue tetrazolium chloride (NBT, a stock solution prepared in 70% dimethylformamide). The band intensities were quantified using Fiji software.

### His-HupS acetylation and deacetylation *in vitro*

For the *in vitro* nonenzymatic acetylation reaction, 1.5 ml of a reaction mixture containing 2.25 mg of purified His-HupS, 100 mM lithium potassium acetyl phosphate (Sigma−Aldrich, US), 10 mM MgCl_2_, 50 mM Tris–HCl pH 8.0 and 300 mM NaCl was incubated at 37°C for 1 h. After incubation, the volume of the reaction mixture was adjusted to 2.5 ml with buffer C (50 mM Tris–HCl pH 8.0, 300 mM NaCl). Acetyl phosphate (AcP) was then removed using filtration on a PD-10 column equilibrated with buffer C. In parallel, a control, nonacetylated His-HupS was prepared following the same protocol but omitting the acetyl phosphate in the reaction mixture. *In vitro*, nonenzymatic His-HupS acetylation was confirmed using 1 μg of acetylated or nonacetylated protein applied directly on a nitrocellulose membrane (dot-blot assay). Protein detection was subsequently performed using the Western blotting protocol described earlier using mouse monoclonal anti-HisTag antibody for loading control and rabbit polyclonal anti-AcK antibody for acetyllysine detection.

The *in vitro* deacetylation was performed at 37°C for 6 h in a 20 μl reaction mixture containing 25 mM Na_2_HPO_4_ pH 8.0, 150 mM NaCl, 1 mM NAD^+^, 0.5 mM DTT, 3 μg of purified His-CobB1 protein and 180 ng of acetylated His-HupS protein. The reaction was stopped with 4 μl of 6X SB buffer (375 mM Tris–HCl pH 6.8, 60% glycerol, 12% SDS, 600 mM DTT, 0.06% bromophenol blue) followed by incubation of the sample at 95°C for 5 minutes. After the deacetylation reaction, 10 μl of the sample was analysed in western blotting with anti-His antibodies to detect His-CobB1 and His-HupS proteins, and 10 μl of the sample was analysed with anti-AcK antibodies to detect lysine acetylation.

### Biolayer interferometry

Biolayer interferometry (BLI) was used for three different biotinylated DNA fragments, including 5′ biotinylated double-stranded DNA (one-end biotinylated dsDNA, 300 bp), 5′ and 3′ biotinylated double-stranded DNA (two-ends biotinylated dsDNA, 300 bp) and 5′ biotinylated single-stranded DNA (one-end biotinylated ssDNA). dsDNA fragments were PCR-amplified using one or two 5′-biotinylated oligonucleotides, BLI_*parS*300_FW and BLI_*parS300*_RV (marked with ‘[Btn]’ in Supplementary Table S3), and pUC19A7 plasmid as a template (constructed by ligation of *S. coelicol*or *parAB* operon 500 bp upstream region amplified using H24*parA*SmaRV and *parA*pset oligonucleotides with pUC19 plasmid) and purified from the agarose gel. 5′-Biotinylated single-stranded DNA (ssDNA, 117 nt; Btn-*parS*-oligo) was synthesized by Sigma−Aldrich (US), diluted and directly loaded as described below. The affinity measurements were performed with an Octet R2 Protein Analysis System (Sartorius, Germany) equipped with Octet High Precision Streptavidin 2.0 SAX2 Biosensors (Sartorius, Germany). In the first step, the streptavidin-coated biosensor was hydrated for 15 minutes on the bench in association buffer (100 mM Tris–HCl pH 8.0, 200 mM NaCl, 1 mM MgCl_2_, 0.005% Tween-20, 0.1 mg/ml BSA). Then, the sensors were mounted onto the Octet R2 Protein Analysis System and calibrated for 60 seconds in association buffer, followed by loading 150 ng of the biotinylated DNA fragments diluted in association buffer for 5 minutes. The association step (450 s) was then established by dipping the biosensors in association buffer containing a broad range of His-HupS concentrations (0–100 nM) in a final volume of 200 μl dispensed in a 96-microwell black plate (Greiner Bio-One, Austria). The His-HupS protein dissociation was subsequently monitored for 180 s in protein-free association buffer. The analyses were performed in cycles of His-HupS association and dissociation. Between each cycle, the biosensor tips were washed for 60 s with association buffer, regenerated for 5 minutes with regeneration buffer (100 mM Tris–HCl pH 8.0, 200 mM NaCl, 1 mM MgCl_2_, 0.005% Tween-20, 0.1% SDS), and equilibrated for 5 min with association buffer. All BLI experiments were performed at 30°C. The data were collected and analysed using Octet Data Analysis software version 11.1. The dissociation constants (*K*_D_) were calculated by fitting the data to the log-logistic model using R Studio software.

### Electrophoretic mobility shift assay (EMSA)

For the EMSA, His-HupS protein (protein concentrations 0–1.2 μM) in EMSA buffer (50 mM Tris–HCl pH 8.0, 150 mM NaCl, 0.02% Tween-20, 10 mM Mg(CH₃COO)₂, 2 μg/μl BSA, 5% glycerol) was incubated for 20 min at room temperature with 200 ng of pUC19A7 plasmid—negatively supercoiled or linear (*XbaI*-digested plasmid purified from agarose gel). The samples were then resolved in 0.8% agarose in Tris-borate-EDTA (TBE buffer (39)) for 14–16 h at low voltage (2 V/cm). The plasmid DNA was visualized by staining the gel in 0.5 μg/ml ethidium bromide solution in TBE buffer for 30 min at room temperature. The stained agarose gel was then analysed in UV light.

### Pulldown analysis

For the HupS-FLAG pulldown assay, 0.05 U of spores of the *S. venezuelae* wild-type and *hupS*-*FLAG* strains (TM015 strain (18)) were used to inoculate 50 ml liquid MYM medium cultures grown at 30°C with shaking (180 rpm) for 13, 17 and 21 h. At each time point, the optical density of the cultures was measured. The cells were collected by centrifugation (5000 g, 10 min, 4°C), washed twice with wash buffer (50 mM Tris–HCl pH 8.0, 250 mM NaCl), and resuspended in 10 ml of lysis buffer (50 mM Tris–HCl pH 8.0, 250 mM NaCl) supplemented with Pierce™ Protease Inhibitor Tablets (Thermo Fisher Scientific, US). The cells were disrupted by sonication, and the obtained cell lysates were clarified by centrifugation (10 000 g, 15 min, 4°C) followed by the filtering of the supernatant through a 0.45 μm pore filter. The clarified cell lysate was supplemented with Tween-20 (Carl Roth, Germany) to a final concentration of 0.05% and incubated overnight with constant tube rotation at 4°C with 50 μl of magnetic beads coated with anti-FLAG® BioM2 antibody (Sigma Aldrich, USA). The next day, the magnetic beads were collected and washed three times with 1 ml of wash buffer. To elute bound protein, the magnetic beads were incubated in SDS-elution buffer (62 mM Tris–HCl pH 6.8, 2% SDS 10% glycerol, 0.01% bromophenol blue) at 95°C for 5 min (for Western blotting). The eluted proteins were analysed by Western blotting using mouse monoclonal anti-FLAG antibodies (for HupS-FLAG detection) or rabbit polyclonal anti-AcK antibodies followed by incubation with HRP-conjugated anti-mouse IgG or anti-rabbit IgG secondary antibodies, respectively, according to the standard western blotting protocol described earlier.

### Intact protein LC−MS

The acetylated and nonacetylated His-HupS samples were concentrated and initially desalted by overnight acetone precipitation at 4°C. The next day, the protein pellet was washed three times with ice-cold acetone and subsequently resuspended in a 0.1% formic acid solution. The total His-HupS mass analysis was carried out with the use of the M-Class Acquity UPLC system coupled to the Synapt XS high-resolution mass spectrometer (Waters Corporation, US) equipped with the Zspray LockSpray II ion source utilizing a 125 μm OD steel emitter. Approximately 7.5 and 75 pmol of the protein were injected for the nonacetylated and acetylated His-HupS samples, respectively. On the system, the samples were further desalted and chromatofocused on a nanoEase M/Z BEH C4 300 Å, 5 μm, 300 μm × 50 mm trap column, while a nanoEase M/Z BEH300 C4 300 Å, 1.7 μm, 300 μm × 100 mm analytical column was employed for chromatographic separation with an eight-minute 10% to 80% acetonitrile linear gradient at a 10 μl/minute flow rate. The columns’ compartment temperature was kept at 80°C. MS data acquisition was collected at 1 scan/s through a 300–3000 *m*/*z* range in positive polarity and TOF resolution mode. Source conditions were as follows: capillary voltage: 3 kV, sampling cone: 40 V, source offset: 15 V, source temperature: 150°C, desolvation gas temperature: 550°C, desolvation gas flow: 800 l/h, cone gas flow: 30 l/h, nebulizer gas pressure: 5.5 bar. Glufibrinopeptide B solution was acquired in the reference function, and the correction was applied post-acquisition. The raw data were processed and analysed using MassLynx V4.2 software. The protein peak from each analysis was integrated, and the combined spectra were background subtracted and then deconvoluted using the MaxEnt1 algorithm.

### Bottom-up LC–MS

For the proteome-scale analysis, the wild-type (WT) and the Δ*hupS* (AKO200) strains grew for 21 h in liquid MYM medium as described earlier for the pulldown experiment. The mycelium was collected and washed twice with 50 mM Tris–HCl buffer pH 7.2. The cell lysate was prepared in 50 mM Tris–HCl buffer pH 7.2, 8 M urea, 0.2% sodium deoxycholate. 10 μg of total protein was denatured for 30 min at 50°C with an addition of 5 mM DTT. Urea concentration was then lowered to 2 M via dilution with 50 mM Tris–HCl buffer pH 7.2, 5 mM DTT, 0.2% sodium deoxycholate solution, and 100 ng of trypsin (EMS0006, Sigma-Aldrich, USA) were added for an overnight digestion at 30°C. The next day the sample was acidified by the addition of formic acid to a final concentration of 0.5%, incubated for 30 min at 37°C, and then centrifuged for 15 min at 15 000 g. The supernatant was further cleaned up using the STAGE tip (42), from which the peptides were eluted using 80% acetonitrile (ACN), and 0.1% formic acid solution. The organic solvent was then removed using the SpeedVac, and the peptide pellet was resuspended in 0.1% formic acid.

For the HupS-FLAG analysis, the wild-type (WT) and *hupS*-*flag* complementation (TM015) strains grew for 21 h in a liquid MYM medium, and HupS-FLAG purification followed the procedure described earlier for the pulldown experiment. The eluted proteins were resolved in SDS-PAGE electrophoresis and CBB-stained. The sections corresponding to the molecular weight of HupS were then excised, and further destained for 30 min at 37°C in 40% ACN, 200 mM ammonium bicarbonate (ABC). The solution was then aspirated, the gel piece crushed with a pipette tip, and dehydrated by another round of incubation in fresh 40% ACN, 200 mM ABC followed by solution aspiration, and its complete removal using a SpeedVac. Dried gel pieces were rehydrated for 45 min at 4°C in 10 μl of 9% ACN, 50 mM ABC, and 5 mM DTT containing 100 ng of trypsin (EMS0006, Sigma-Aldrich, USA). Trypsin-loaded gel samples were then further hydrated via an addition of 30 μl of 9% ACN, 50 mM ABC and 5 mM DTT and then left for overnight digestion at 30°C. The next day the samples were briefly centrifuged, and the peptide-containing supernatant was transferred into a fresh tube. An additional peptide extraction step was carried out by incubation of the sedimented gel pieces for 30 min at 37°C in 30 μl of 9% ACN, 50 mM ABC, and 5 mM DTT. The recovery solutions were pooled, volatiles were removed by SpeedVac, and the obtained pellet was reconstituted in 40 μl of 0.1% formic acid.

Analyses were carried out with the use of the M-Class Acquity UPLC system coupled to the Synapt XS high-resolution mass spectrometer as described above. The amount corresponding to 800 ng of total input protein was loaded for the lysate samples, and 1 μl of a 4× dilution of the final sample was used for the pull-downs. Chromatofocusing was carried out with the nanoEase M/Z Symmetry C18 100 Å, 5 μm, 180 μm x 20 mm trap column while the nanoEase M/Z HSS T3 C18 100 Å, 1.8μm, 75 μm x 150 mm analytical column was employed for the chromatographic separation with 90 min (lysates) or 60 min (pulldown samples) 5% to 35% acetonitrile linear gradient at a 300 nl/min flow rate. The columns’ compartment temperature was kept at 40°C. Data were collected in ultra-definition ion mobility DIA (UDMS^E^) at 0.6 scan/s through a 50–2000 *m*/*z* range in positive polarity and TOF resolution mode. An ion's drift-time-adjusted collision energy ramp, specifically determined for the instrument, was applied on the transfer cell (43). Source conditions were as follows; capillary voltage: 2.5–2.6 kV, sampling cone: 40 V, source offset: 15 V, source temperature: 100°C, cone gas flow: 40 l/h, purge gas flow: 0.6 l/h, NanoFlow gas pressure: 0.5—1.9 bar. A (Glu1)-Fibrinopeptide B solution was acquired in the reference function, and the correction was applied post-acquisition. Before processing, lysates’ raw data were noise reduced with an ion count threshold of 10.

Data processing was performed using the Protein Lynx Global Server (PLGS) v3.0.3 software. For the deconvolution, the low and high energy ion threshold counts were set to 135 and 30, respectively, and the chromatographic and TOF peak detection parameters were set to Apex3D engine's autovalues. Obtained deconvoluted spectral data were merged from duplicates (cell lysate) or triplicates (pulldown), and searched via the Ion Accounting algorithm against either a *S. venezuelae* protein sequence databank (UP000006854) to which the trypsin sequence accession was appended (cell lysate) or an alike databank in which the wild-type HupS accession (F2R5C9) was substituted with the recombinant HupS-FLAG accession (pulldown). The set search parameters were as follows; peptide mass tolerance: 25 ppm; fragment mass tolerance: 25 ppm; min. fragment ion matches per peptide: 1 (lysates), 3 (pull-downs); min. fragment ion matches per protein: 3 (cell lysate), 7 (pulldown); min. peptide matches per protein: 1; max. protein mass: 1 MDa; digest reagent: Trypsin (cut after K and/or R, but not before P); Allowed missed cleavages: 10; fixed modification: none Variable modification: Oxidation of M (+15.99491 Da, applies to M sidechain); Enriched variable modification (in order to enforce searching in pass one): Acetylation of K (+42.01056 Da, applies to K sidechain), FDR: 1%.

The obtained data were phenotype-compliant with no HupS hit in the *ΔhupS* strain sample (with 1024 protein groups identified total), as well as no HupS hit in the wild-type anti-FLAG pull down (6 protein groups total). Consistently HupS was identified in the wild-type strain sample (819 protein groups total), and the TM015 anti-FLAG pull down (12 protein groups total). The former two samples were therefore used as false positive controls in confirming HupS acetylated peptides. HupS accession peptide tables from both samples were manually analyzed and peptides with an acetyllysine (AcK) modification were validated based on generally known characteristics of AcK bearing peptides, such as a non-possibility of trypsin cleavage at the C-terminus of an acetylated lysine and a general upshift of retention time between an acetylated and a non-acetylated peptide of same kind (44,45). Peptides not complying were assumed as false positives. Further validation was based on the quality assessment of the MS2 spectral evidence and the peptide's absence in the unmatched spectral data from corresponding control samples. Peptides with poor MS2 matches (ions within noise level, abnormal precursor/fragment abundance ratio, etc.), as well as peptides, whose ions were found in controls (based on MH+, RT, Drift and intensity comparison), were rejected. In the pulldown sample an expected higher count of MS2 fragment matches was obtained (due to HupS enrichment) enabling precise identification down to a specific HupS lysine residue. Based on the pulldown results, assumptions were made for peptides identified in the lysate sample if the MS2 evidence was not unambiguous in terms of AcK residue assignment.

### RNA isolation and RT−qPCR

For RT−qPCR analyses, the *S. venezuelae* wild-type strain was inoculated with 0.05 U of spores and cultivated in 5 ml liquid MYM medium for 8–25 h as described earlier. RNA from mycelia was isolated using the GeneJET RNA isolation kit (Thermo Fisher Scientific, USA), according to the manufacturer's protocol. However, the lysozyme concentration in the suspension buffer was increased to 10 mg/ml to improve the yield of mycelium lysis. The isolated RNA was digested with TURBO DNase I (Invitrogen, United States) and subsequently purified and concentrated using a GeneJET RNA Cleanup kit (Thermo Fisher Scientific, USA). 500 ng of total DNA-free RNA were used for cDNA synthesis using the Maxima First Strand cDNA synthesis kit (Thermo Fisher Scientific, USA). The original manufacturer's protocol was modified for GC-rich *S. venezuel*ae transcripts by increasing the temperature of the first-strand synthesis to 65°C and extending the synthesis time to 30 min. Next, the cDNA sample was diluted to 100 μl with miliQ water, and 2 μl was directly used for quantitative qPCR. qPCR was performed using PowerUP SYBR Green Master Mix (Applied Biosystems, USA). The relative levels of the *cobB1* transcript were compared using the comparative ΔΔCt method and the *hrdB* transcripts as the endogenous control. The oligonucleotides are listed in Supplementary Table S3. Transcripts per million (TPM) were obtained from the raw RNA-Seq data available in Array Express (EMBL-EBI) database under accession numbers E-MTAB-13607 (46) and E-MTAB-13911 (47). The complete description of the RNA-Seq protocols including data acquisition and sample collection is attached to the deposited data.

### Glutaraldehyde crosslinking

A glutaraldehyde crosslinking reaction was performed in 15 μl samples containing 12.5 mM Tris–HCl pH 8.0, 15% glycerol, 75 mM NaCl, and proteins: 4 μM His-HupS, 4 μM HupA or both. For crosslinking, glutaraldehyde was added to a final concentration of 0.005, 0.015, or 0.03%. For the control reaction, 5 μl of miliQ water was added instead of glutaraldehyde. Then, the samples were incubated at 37°C for 30 min. After incubation, the proteins were denatured by adding 4 μl of 6X sample buffer (375 mM Tris–HCl pH 6.8, 60% glycerol, 12% SDS, 600 mM DTT, 0.06% bromophenol blue) followed by incubation at 95°C for 5 min. The samples were resolved by standard Laemmli acrylamide gel electrophoresis (SDS−PAGE) followed by protein CBB-staining with InstantBlue^®^ Coomassie Protein Stain (Abcam, UK).

### Fluorescence microscopy

For the snapshot analysis of nucleoid areas, spores of *S. venezuelae* strains were diluted in sterile miliQ water and cultured for 22–24 h on SFM agar plates with sterile coverslips inserted at a 45° angle. Next, the mycelia on the coverslips were fixed by washing twice with absolute methanol. The nucleoid and cell wall were stained for 30 min at room temperature with 7-amino-actinomycin D (1 mg/ml 7-AAD in DMSO, Invitrogen, USA) diluted 1:300 and wheat germ agglutinin conjugated with Alexa-Fluor-350 (1 mg/ml WGA-AF_350_ in DMSO, Invitrogen, USA) diluted 1:200 in filtered PBS buffer. In the next step, the mycelium was washed four times with filtered PBS buffer, and the coverslips were mounted on microscopic glass slides using 50% glycerol solution in filtered PBS buffer. The mycelium was imaged using a Leica DM6 fluorescence microscope equipped with a 100×/1.4 oil objective in differential interference contrast (DIC), DAPI and mCherry channels. The nucleoid area was analysed in aerial hyphae with visible septa staining (prespores) with custom Fiji software protocols. The boxplots showing the distribution of nucleoid areas and statistical analysis were obtained using an *EDA* tool (the code is available at https://github.com/astrzalka/EDA). The Wilcoxon test was used to determine the statistical significance of the differences between populations.

### Single-particle tracking

For single-particle tracking (SPT), *S. venezuelae hupS-halotag* strains (AZ01 and JD14) were cultured for 23 h in 1 ml of liquid MYM medium supplemented with 5 nM HaloTag® ligand TMRDirect™ (Promega, USA). Immediately before imaging, mycelia were washed three times with filtered PBS buffer and spread onto agar pads (1.1% low gelling temperature; Sigma-Aldrich, USA) poured into Gene Frames (chamber 1.0 × 1.0 cm; Thermo Fisher Scientific, USA) and covered with an 18 × 18 mm coverslip (High Precision Microscope Cover Glasses, Carl Roth, Germany). Before sample preparation, microscope slides and coverslips were prepared by overnight incubation in 1 M KOH solution and subsequent drying with nitrogen flow. SPT was carried out at 30°C using a Zeiss Elyra 7 inverted microscope equipped with an Andor iXon DU 897 electron multiplying charge-coupled device (EMCCD) camera and an alpha Plan-Apochromat 100×/1.46 Oil DIC M27 Elyra objective in combination with laser lines HR DPSS 561–500 200 mW 561 nm. The Z-axis was stabilized via the ‘definite focus’ system. The samples were prebleached, and the images were recorded using a 561 nm laser (80% laser power, EMCCD gain of 100) with an exposure time of 20 ms in HILO illumination mode (TIRF Angle 47.26°). For each position, 10000 frames were collected. SPT was performed using Fiji, TrackMate and SMTracker software (48,49). Cell segmentation was performed using Fiji and Oufti software (50). First, for each strain, the frequency of jump distances (JD) between consecutive frames was plotted. Then, the Squared Displacement (SQD) or Gaussian Mixture Model (GMM) analyses using SMTracker software were performed independently to distinguish between mobile and immobile HupS-HaloTag molecules. Whereas the SQD method uses the cumulative probability of square displacements, the GMM method uses the displacement of molecules between consecutive frames to fit two Gaussian models representing DNA-bound and free proteins. Both methods allowed independently for the calculation of the apparent diffusion coefficients and the relative fractions of two diffusive states of the HupS-HaloTag protein. To calculate the average residence time [ms] for molecules bound to DNA the stationary localization analysis (SLA) with SMTracker software was used to determine the number of events when the molecule remained within a specified radius and to calculate the average residence time. Due to cell confinement and motion blurring, D* is an apparent diffusion coefficient. All experiments were carried out as two independent biological replicates and combined subsequently for GMM or SQD analyses as described above.

## Results

### HupS protein is modified posttranslationally by lysine acetylation

**Figure 1.**
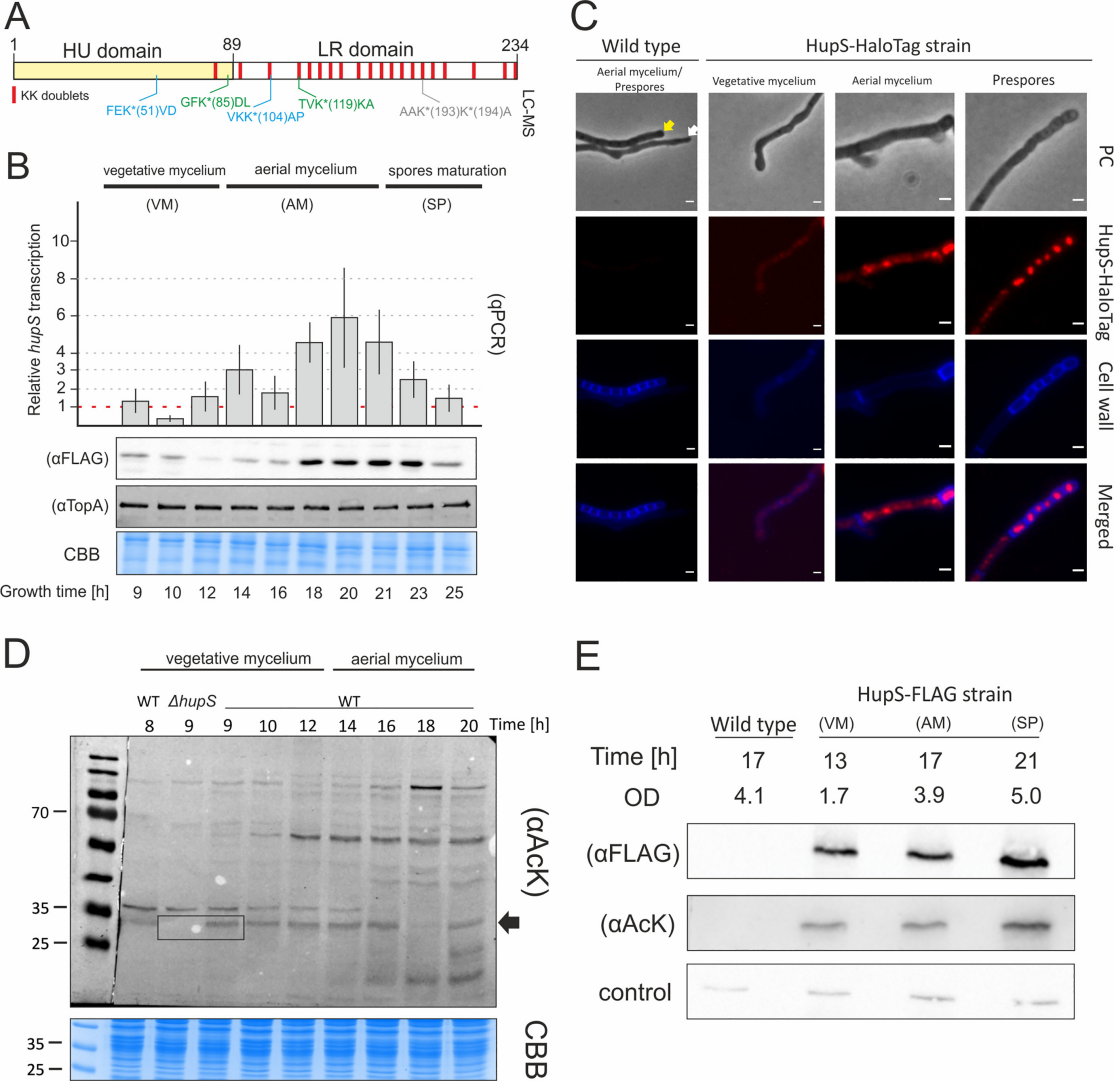
HupS levels upregulation and its acetylation during *S. venezuelae* growth and development. (**A**) The scheme of *S. venezuelae* HupS protein organisation. The HU domain (amino acids 1–89) homologous to the *E. coli* HU protein is marked in yellow, whereas the C-terminal domain (amino acids 90–234) rich in lysine repeats (LR domain) is marked in white. The lysine repeats (KK doublets) are marked in red. The acetylated lysine residues (K*) identified in the HupS-FLAG pulldown experiment (marked in blue) or in the untagged HupS protein during global proteome studies (marked in grey), or in both LC-MS analyses (marked in green), are indicated. The HupS or HupS-FLAG lysine modifications were studied in *S. venezuelae* culture growing for 21 h in liquid MYM medium. (**B**) Analysis of *hupS* transcript (in wild-type strain, WT) and HupS-FLAG protein levels (in *hupS-flag* strain, TM015) during the *S. venezuelae* life cycle (8–25 h of growth in MYM medium). The particular growth stages, including vegetative growth (VM), aerial mycelium formation (AM), and spore maturation (SP), are marked. The *hupS* transcripts were quantified in relation to their levels in 8 hour of growth (estimated as 1, red line). HupS-FLAG protein levels were quantified at corresponding time points using anti-FLAG antibodies (αFLAG). TopA levels quantified using anti-TopA serum (αTopA) served as an internal control for the experiment. The loading control (CBB-stained acrylamide gel) is shown below. (**C**) Localization of HupS-HaloTag (red) at subsequent *S. venezuelae* developmental stages (in the *hupS-halotag* strain, AZ01). The cell wall was visualized using wheat germ agglutinin (WGA) conjugated with Alexa Fluor 350 (blue). The hyphal morphology was visualized using phase contrast (PC). Examples of aerial mycelia and chains of prespores are marked with white and yellow arrows, respectively. Scale bar: 1 μm. (**D**) Western blot detection of protein lysine acetylation in the cell lysates of the wild-type *S. venezuelae* strain cultured in liquid MYM. As the control, the lysate obtained from the *hupS* deletion strain (in 9 hour of growth, *ΔhupS* strain, AKO200) was used. The putative band corresponding to the HupS protein is marked with a black arrow. The loading control (CBB-stained acrylamide gel) is shown below. The 25, 35 and 70 kDa bands of the protein molecular wemultight ladder are marked. (**E**) The pulldown of HupS-FLAG protein from *S. venezuelae* cell lysates obtained at different developmental stages (vegetative (VG) and aerial (AM) mycelium, or spore maturation (SP)) of *S. venezuelae* (*hupS-flag* strain, TM015) growing for 13, 17 and 21 h in liquid MYM medium. The optical density of the culture is indicated for each growth phase. The HupS-FLAG protein levels were detected using anti-FLAG antibodies (αFLAG). Lysine acetylation was detected using anti-AcK antibodies (αAcK). The co-eluted heavy chain of IgG antibodies detected by anti-mouse IgG served as a loading control.

The *S. venezuelae* HupS protein (234 aa; 23.8 kDa), encoded by the *vnz_25 950* gene (*hupS*), is composed of two domains. The N-terminal domain shows homology to HU proteins, whereas the 144 amino acid C-terminal fragment (90–234 aa) resembles the histone H2B tail and contains 43 lysine residues, and 38 of them are accumulated as KK doublets (Figure 1A and Supplementary Figure S1A) (18). Earlier studies have shown that in *S. coelicolor*, HupS levels increase during sporogenic development due to the transcriptional upregulation of the *hupS* gene (19). To confirm whether the same *hupS* transcriptional upregulation occurs during the development of *S. venezuelae*, we measured the levels of the *hupS* transcript during differentiation of the wild-type strain, and we monitored HupS-FLAG and HupS-HaloTag protein levels using strains expressing *hupS-flag* or *hupS-halotag* genes, (TM015 and AZ01 respectively). Both genes encoding fusion proteins were delivered *in trans* under the control of the native *hupS* promoter (Supplementary Table S2). Our previous studies (18), also reinforced here (Figure 5C and E), showed that HupS C-terminal fusion with the FLAG-tag complemented the phenotype of *S. venezuelae hupS* deletion strain, confirming the functionality of HupS-FLAG recombinant protein.

Analyses of *hupS* transcript levels between 9th and 25th hour of growth confirmed that, the *hupS* gene is strongly activated during S*. venezuelae* differentiation, reaching the maximum transcript levels prior to sporulation (up to 6-fold in relation to the vegetative phase) and gradually decreasing during spore maturation (Figure 1B). The increase of HupS-FLAG protein levels detected with anti-FLAG antibodies correlated with *hupS* gene upregulation during *S. venezuelae* sporogenic development, although the protein levels remained constant throughout most of the spore maturation phase (with the exception of 25 h of growth when we detected a rapid decrease in HupS-FLAG levels; Figure 1B). This observation corroborates the microscopy observation of the HupS-HaloTag signal during development. In *S. venezuelae* vegetative hyphae, HupS-HaloTag fluorescence was relatively low but increased strongly in the aerial mycelium and remained elevated during spore maturation (Figure 1C). Our analyses confirmed the previous reports (18,19) of the HupS upregulation during sporogenic development and its localisation in *Streptomyces* spores (Figures 1B and C).

Next, we investigated whether HupS may be regulated not only by the transcriptional activation of the *hupS* gene (Figure 1B) but also posttranslationally by reversible lysine acetylation, as found previously for its *M. tuberculosis* homologue (HupB) (30) or the lysine-rich tail of eukaryotic histone H2B (51). First, we assessed global protein acetylation (acetylome) in lysates obtained from *S. venezuelae* wild-type and *hupS* null mutant strain cultures. Western blotting with anti-AcK polyclonal antibodies showed that the total signal from acetylated proteins increased in the wild-type *S. venezuelae* strain during development progression. Moreover, one of the acetylated proteins (with a molecular mass slightly below 35 kDa) was present in the wild-type strain but not in the acetylome of the *hupS* deletion strain, while other acetylated proteins were still detectable in both strains at comparable levels. Thus, we speculated that this protein may correspond to HupS (Figure 1D).

To confirm HupS acetylation *in vivo*, we used the *S. venezuelae hupS-flag* strain to purify the recombinant HupS-FLAG protein (with anti-FLAG antibodies immobilized on magnetic beads) from the cell lysates collected at three developmental stages: vegetative growth (VM), aerial mycelium formation (AM) and the spore maturation phase (SP). Subsequently, the purified HupS-FLAG protein was detected with anti-FLAG and anti-AcK antibodies to detect the HupS-FLAG in the eluent and HupS-FLAG lysine acetylation, respectively. The lysine-acetylated protein was detected at all three developmental stages, suggesting that HupS-FLAG is constantly acetylated throughout the *S. venezuelae* life cycle (Figure 1E).

We also attempted to identify HupS acetylation sites *in vivo*. For that, we used LC–MS analyses based on untagged HupS protein (global proteome analysis of the WT type strain) or purified HupS-FLAG (pulled down from TM015 strain lysate). We identified two acetylated lysine residues positioned within the N-terminal HU domain (K51 and K85) and four acetylated lysine residues located within the LR domain (K104, K119, K193 or K194). Two of them (K85 and K119) were detected in both analyses (Figure 1A and Supplementary Figure S2A) confirming HupS acetylation *in vivo*. Unfortunately, the low peptide coverage within the C-terminal domain strongly limited the detection of other potential acetylation sites within the LR domain (Supplementary Figure S2A). However, the western blotting (with anti-FLAG antibodies) identified the polypeptides, which co-purified with the intact HupS-FLAG. Their molecular weights, at least 10 kDa lower than the intact protein, suggested partial degradation of the N-terminal fragment of the HupS-FLAG protein. These peptides were also detected using anti-AcK antibodies indicating lysine acetylation within the LR domain (Supplementary Figure S2B). Thus, even though LC–MS studies delivered limited information concerning acetylation of the LR domain, our analyses reinforced by the Western blot confirmed the lysine acetylation within either the N- and C-terminal domains of HupS.

In summary, we confirmed that in *S. venezuelae, hupS* is regulated at the transcriptional level, and its expression increases during sporogenic development. Moreover, we provided the first evidence that HupS-FLAG, similar to its mycobacterial homologue HupB, is acetylated on lysine residues *in vivo*.

### Lysine acetylation affects the DNA affinity of HupS *in vitro*

**Figure 2.**
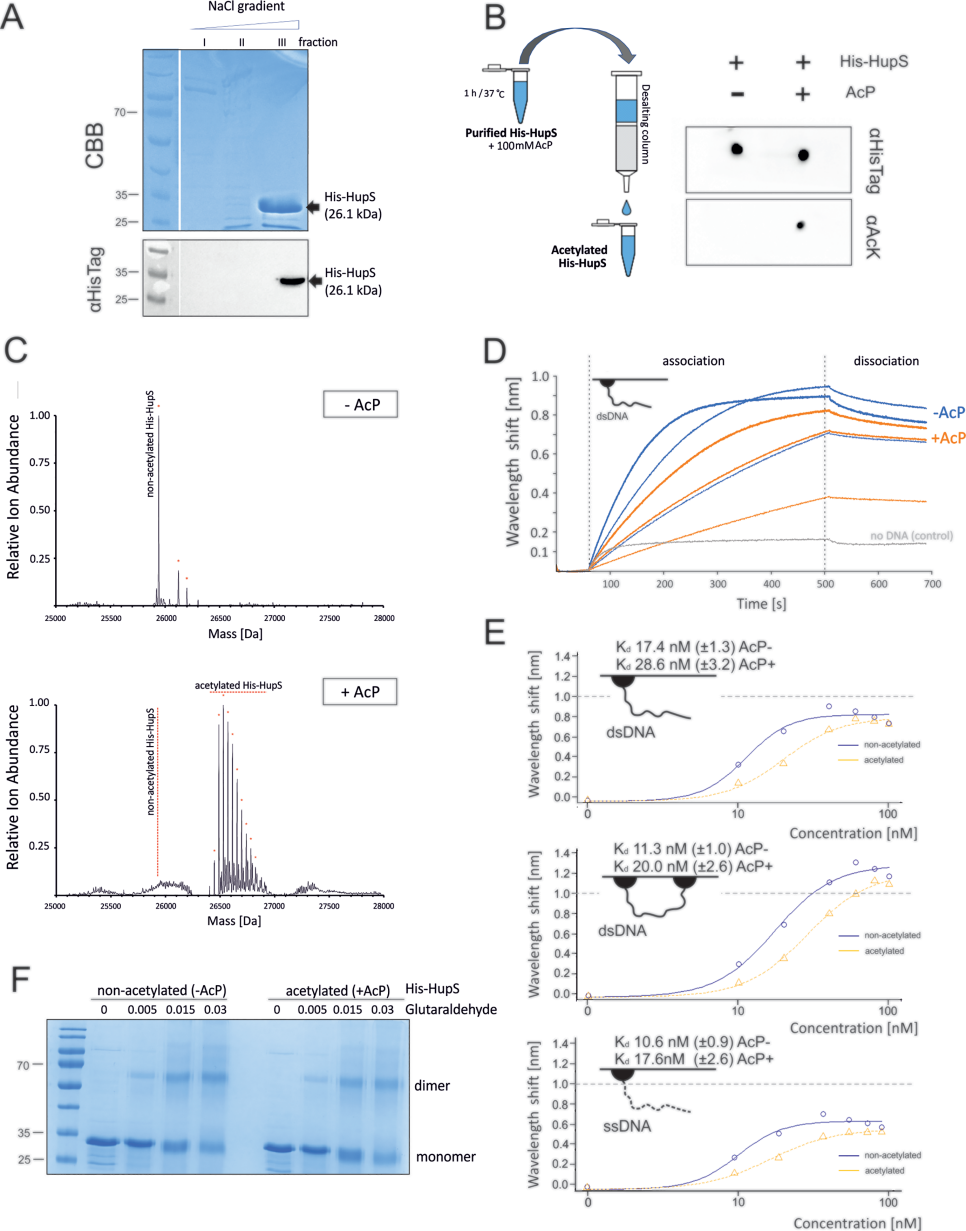
Acetylation of recombinant His-HupS and its impact on protein−DNA interactions *in vitro*. (**A**) His-HupS (26.1 kDa) purification using ion exchange chromatography. His-HupS protein (black arrow) was eluted using a 3-step NaCl gradient (250 mM (I), 500 mM (II), and 800 mM (III)) and analysed using SDS−PAGE electrophoresis followed by CBB staining or Western blotting using anti-HisTag antibodies. (**B**) Left panel: scheme of His-HupS acetylation *in vitro* in the presence of 100 mM acetyl phosphate (AcP) followed by protein desalting. Right panel: detection of His-HupS using a dot-blot assay with anti-HisTag (for protein detection, αHisTag) or anti-acetyllysine antibodies (for lysine acetylation detection, αAcK). (**C**) LC−MS detection of non-trypsin-digested acetylated (+AcP) and nonacetylated (-AcP) recombinant His-HupS protein. (**D**) Binding of acetylated (+AcP, orange) and nonacetylated (-AcP, blue) His-HupS (protein concentrations: 20, 40 and 60 nM) to one-end biotinylated double-stranded DNA (dsDNA, 300 kb) measured using biolayer interferometry (BLI). The binding of nonacetylated His-HupS (40 nM) to a DNA-free sensor (grey) served as a control. The HupS-His association and dissociation steps are marked in the scheme. (**E**) The analysis of acetylated and nonacetylated His-HupS protein affinity to 3 types of DNA substrates (one- or two-end biotinylated double-stranded DNA, dsDNA, 300 bp, or one-end biotinylated single-stranded DNA, ssDNA, 117 nt) performed in a broad range of His-HupS protein concentrations (0–100 nM). The quantified His-HupS dissociation constants (K_d_) calculated for particular DNA substrates are shown above each plot. (**F**) Glutaraldehyde crosslinking of acetylated (+AcP) and nonacetylated (–AcP) His-HupS. The migration of His-HupS monomers and dimers was analysed using SDS-PAGE electrophoresis. The 25, 35 and 70 kDa bands of the protein molecular weight ladder are marked on the left. The glutaraldehyde concentrations (%) are indicated.

Having confirmed HupS acetylation *in vivo*, we set out to determine the impact of this HupS modification on its DNA binding. To study the influence of lysine acetylation on HupS-DNA interactions, we overproduced His-HupS recombinant protein (26.1 kDa) in *E. coli*, and purified it using ion exchange chromatography and salt gradient fractionation (Figure 2A). Taking into account the high positive charge of the nonacetylated HupS protein (theoretical pI = 11.14), which is lowered when the protein is acetylated, this simplified, one-step procedure delivered nonacetylated His-HupS (or acetylated at a low level). Although the purified recombinant protein migrated slower (slightly below 35.0 kDa) in SDS-PAGE gel than suggested by its molecular weight prediction (26.1 kDa), anti-HisTag antibodies detected His-tagged HupS protein in the collected fraction III (Figure 2A), while the anti-AcK antibodies and LC−MS analysis did not detect its acetylation (Figures 2B and C). In the next step, we acetylated the His-HupS protein *in vitro* (Figure 2B) in the presence of 100 mM acetyl phosphate (AcP) as an acetyl group donor (52,53). The acetylation of the His-HupS protein (+AcP) compared to the nonacetylated protein (–AcP) was verified using an anti-AcK antibodies (Figure 2B) and LC−MS analysis of the nontrypsinized His-HupS protein (Figure 2C). The acetylation *in vitro* delivered the multiacetylated His-HupS protein carrying from 12 to up to 21 modified lysine residues per a single His-HupS molecule with a dominant fraction of the proteins modified from 13 to 17 times per single His-HupS molecule (Supplementary Figure S2C).

To study the impact of multiple acetylation of His-HupS protein on its DNA binding, we used biolayer interferometry (BLI) and one-end biotinylated double-stranded DNA fragments (300-kb dsDNA). The comparative DNA binding analysis of two His-HupS variants, acetylated (+AcP) and nonacetylated (–AcP), showed that the nonacetylated His-HupS protein associated more rapidly with DNA than its acetylated variant at all tested protein concentrations (20–60 nM) (Figure 2D). The subsequent quantification of the dissociation constants (*K*_d_) for the acetylated and nonacetylated proteins showed a decreased DNA affinity of lysine-acetylated His-HupS (*K*_d_ = 28.6 ± 3.2 nM) in comparison to its nonacetylated variant (*K*_d_ = 17.4 ± 1.3 nM) (Figure 2E). When dsDNA fragments were immobilized using both ends (two-ends immobilized dsDNA), His-HupS affinity to DNA was higher than when the DNA was immobilized using only one end. Nevertheless, acetylated His-HupS had an affinity lower than that of the acetylated protein (*K*_d_ = 11.3 ± 1.0 nM and *K*_d_ = 20.0 ± 2.6 nM for nonacetylated and acetylated variants, respectively) (Figure 2E). Analysis of a single-stranded (117 nt), one-end immobilized oligonucleotide showed that His-HupS was also able to bind single-stranded DNA (ssDNA) with comparable affinity as for double-stranded, two-end immobilized DNA (*K*_d_ = 10.6 ± 0.9 nM), and the affinity of His-HupS to this substrate also decreased as a result of His-HupS lysine acetylation (*K*_d_ = 17.6 ± 2.6 nM) (Figure 2E). In addition to BLI analyses, we also investigated His-HupS affinity for supercoiled and linearized plasmid DNA using an electrophoretic mobility shift assay (EMSA), showing that the nucleoprotein complex formed more efficiently in the presence of supercoiled plasmid DNA in comparison to the same amount of the linearized plasmid (Supplementary Figure S3). These observations suggested that although HupS has no nucleotide sequence specificity (18), its DNA affinity might depend on the DNA substrate with a preference for ssDNA or supercoiled DNA.

Earlier studies on HU homologues in *E. coli* showed that HUα and HUβ may form dimers (54). It could not be excluded that HupS acetylation disturbs HupS-DNA complex formation by affecting HupS dimerization. Thus, next, we tested whether the lower DNA affinity might be correlated with putative disturbances in protein−protein interactions of the acetylated His-HupS variants. Glutaraldehyde crosslinking followed by SDS−PAGE analysis showed that both the acetylated (AcP+) and nonacetylated (AcP–) His-HupS variants formed high-molecular weight complexes with similar efficiency (Figure 2F). Thus, this observation suggested that the decreased DNA affinity of the acetylated HupS did not result from disturbances in protein−protein interactions but rather was the effect of lowered affinity of HupS monomers and/or dimers for DNA due to lysine acetylation.

In summary, our *in vitro* studies showed that the His-HupS protein bound single- and double-stranded DNA and that multiple lysine acetylation decreased HupS affinity for all tested DNA substrates but did not affect HupS dimerization. Moreover, the *in vitro* studies showed the preference of the HupS protein for single-stranded or supercoiled DNA.

### CobB1 affects HupS acetylation levels *in vitro* and *in vivo*

**Figure 3.**
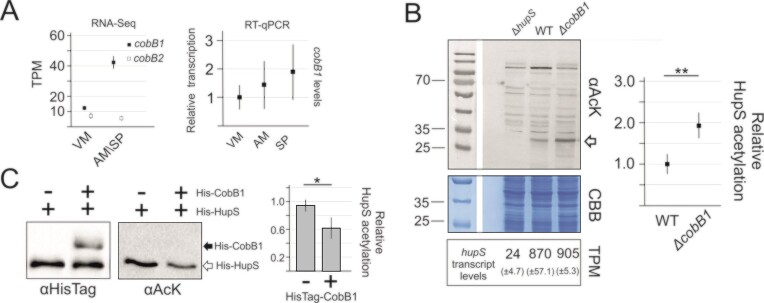
CobB1 protein deacetylase activity *in vivo* and *in vitro*. (**A**) Left panel: *cobB1* and *cobB2* levels (TPM, transcripts per million) based on transcriptome analyses (RNA-Seq data). The analysed *S. venezuelae* developmental stages, vegetative mycelium (VM; 10 h of growth in liquid MYM) and aerial mycelium/spore maturation (AM/SP; 20 h of growth in liquid MYM), are indicated. Right panel: Relative *cobB1* transcript levels quantified in triplicate using RT-qPCR and RNA extracted at different *S. venezuelae* developmental stages; vegetative mycelium (VM), aerial mycelium (AM) and during spores maturation (SP) after 13, 17 and 21 h of growth in liquid MYM, respectively. (**B**) Left panel: Western blotting detection of lysine acetylation in *S. venezuelae* cell lysates obtained from the Δ*hupS* (AKO200), wild-type (WT) and Δ*cobB1* (JD01) strains. The loading control (CBB-stained acrylamide gel) is shown below. The levels of *hupS* transcript (transcripts per million, TPM) in the corresponding strains are shown below. Right panel: HupS acetylation quantified in triplicate in the *ΔcobB1* and wild-type strains. The statistical significance was tested using an unpaired *t* test (*P* value < 0.01, **). (**C**) The activity *in vitro* of recombinant 3 μg His-CobB1 (4.3 μM) protein against 180 ng of multi-lysine-acetylated (13–17 times on average) His-HupS (0.4 μM) after 6 h of incubation at 37°C. The estimated molar ratio of AcK sites/His-CobB1 sites was from 1.2 to 1.6 on average. The His-CobB1 (black arrow) and His-HupS (white arrow) levels were detected using Western blotting and anti-His antibodies (serving as a loading control, left panel, αHisTag). His-HupS acetylation was detected using western blotting and anti-AcK antibodies (αAcK). Right panel: quantification of the His-HupS acetylation signal in the presence of His-CobB1 in triplicate in relation to the control lacking His-CobB1. The statistical significance was tested using an unpaired *t* test (*P* value < 0.05, *). The 25, 35 and 70 kDa bands of the protein molecular weight ladder are marked on the left.

The *S. venezuelae* genome encodes two putative Sir2-like protein deacetylases—CobB1 (*vnz_03 080*) and CobB2 (*vnz_31 025*). Our transcriptomic analyses (46), confirmed subsequently by RT−qPCR, showed that *cobB1* transcript levels increased during *S. venezuelae* differentiation, reaching up to 2-fold elevated levels during aerial mycelium formation or sporogenic differentiation. On the other hand, the *cobB2* gene was poorly transcribed during both vegetative and aerial growth (Figure 3A). Taking into account the levels of both potential deacetylases in relation to the increase in protein acetylation levels during *S. venezuelae* development (Figure 1D), we set out to test whether HupS could be the target for CobB1.

First, we assessed the effect of *cobB1* gene deletion on HupS acetylation *in vivo*. To this end, we constructed *cobB1* and *cobB2* deletion strains and compared the acetylome (using anti-AcK antibodies) in these strains to that of the wild-type *S. venezuelae* and *hupS* deletion strains. Only *cobB1* deletion mildly affected the level of most protein acetylation, with the exception of the HupS protein. The signal corresponding to HupS (observed in the wild-type strain but not detected in the *hupS* deletion background) was up to 2-fold elevated in the *cobB1* deletion background (Figure 3B and Supplementary Figure S4A) while the *hupS* transcript levels remained constant. Moreover, we observed that *cobB1* deletion did not induce *cobB2* gene transcription and, on the other hand, *cobB2* deletion did not affect HupS acetylation levels in contrast to the *cobB1* or double *cobB1/cobB2* deletion strains (Supplementary Figures S4B and C), thus indicating the preferential involvement of CobB1 deacetylase in the regulation of the HupS acetylation state *in vivo*. To gain more insight into the impact of CobB1 on HupS, we also constructed and analysed an *S. venezuelae* strain expressing an additional copy of the *cobB1* gene under the control of the constitutive *ermE* promoter (*his*-*cobB1*↑ strain). Analyses of the acetylome in the *cobB1* overexpressing strain (*his*-*cobB1*↑) showed decreased lysine acetylation signals of several proteins, including the HupS protein, indicating a more pronounced effect of *cobB1* overexpression than its deletion (Supplementary Figure S5). Since the *S. venezuelae* acetylome was only partially affected by *cobB1* deletion or overexpression, this result suggests that the CobB1 deacetylase may show some specificity towards acetylated substrates, and among a limited number of protein substrates, acetylated HupS can be recognized and processed preferentially by CobB1. To confirm the activity of the CobB1 protein as a HupS deacetylase *in vitro*, we overproduced His-CobB1 (35.0 kDa) recombinant protein in *E. coli* and purified it (Supplementary Figure S6A). Subsequently, we used the *in vitro* acetylated His-HupS protein as a substrate for His-CobB1 deacetylase. We detected decrease in the relative His-HupS acetylation signal (up to 40%) in the presence of purified His-CobB1 and NAD^+^ as the essential cofactor as compared to the control sample in the absence of His-CobB1 (Figure 3C and Supplementary Figure S6B).

In summary, we showed that the levels of *cobB1* transcripts increase during sporogenic *S. venezuelae* development. We confirmed that the lack of CobB1 deacetylase increases HupS acetylation while its overproduction decreases HupS acetylation *in vivo*. Moreover, we showed that *S. venezuelae* CobB1 has NAD^+^-dependent deacetylase activity *in vitro*, recognizing acetylated HupS as a substrate.

### The mobility of HupS molecules *in vivo* is influenced by CobB1 activity

**Figure 4.**
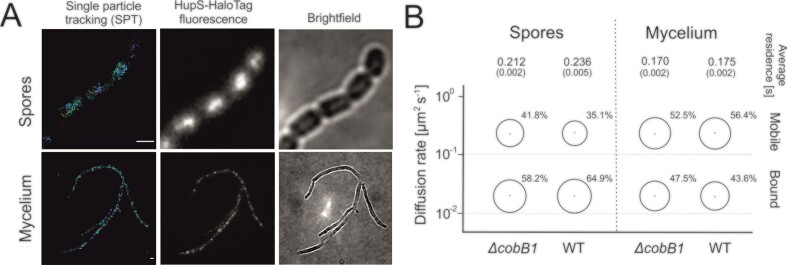
Single-particle tracking (SPT) of HupS-HaloTag. (**A**) Example of SPT tracks collected for HupS-HaloTag protein in *S. venezuelae* spores and mycelia. The HupS-HaloTag tracks are presented in relation to the total HupS-HaloTag fluorescence and the phase contrast (PC) image of the corresponding spores and mycelium. Scale bar: 1 μm. (**B**) The diffusion rates (DR; μm^2^s^−1^) were quantified for the *S. venezuelae* wild-type (WT) and *ΔcobB1* (JD01) strains. The fractions (%) of bound (DR < 10^−1^ μm^2^s^−1^) and mobile (DR > 10^−1^ μm^2^s^−1^) HupS-HaloTag molecules were calculated for *S. venezuelae* mycelium and spores, respectively, using GMM method. The average HupS-HaloTag residence time [s] was calculated for HupS-HaloTag in spores/mycelium in two genetic backgrounds (wild-type and *ΔcobB1*; AZ01 and JD14 strains, respectively).

Based on our above-described observations, we hypothesized that CobB1 activity may affect HupS affinity to DNA in *S. venezuelae* hyphae and spores. To test our hypothesis, we used *S. venezuelae* strains producing HupS-HaloTag protein in the wild-type and *ΔcobB1* background and analysed the mobility of protein molecules by high-resolution microscopy and a single particle tracking (SPT) approach (Figure 4A and Supplementary Figure S7). The earlier described applications of SPT for DNA binding proteins showed that DNA-bound proteins had low mobility, while cytoplasmic, nonbound protein molecules were highly mobile (55–58).

HupS-HaloTag SPT studies showed that in the wild-type strain, the fraction of immobile, presumably DNA-bound protein increased from 43.6% mycelium to 64.9% in spores (measured in 23th hour of growth) (Figure 4B). Moreover, in the wild-type background, the average HupS-HaloTag residence time increased from 0.175 to 0.236 s in mycelium and spores, respectively (Figure 4B). These observations suggest that during sporogenic development, more HupS-HaloTag molecules are associated with DNA or higher DNA compaction restricts HupS-HaloTag mobility. The *cobB1* deletion resulted in a slight increase in the immobile, DNA-bound fraction of HupS-HaloTag in hyphae; however, the average residence time on DNA was similar (0.170 s) in comparison to the wild-type background (0.175 s). The effect of *cobB1* deletion was more prominent in spores and led to an increased fraction of mobile HupS-HaloTag proteins, from 35.1% in the wild-type strain to 41.8% in the *ΔcobB1* strain. Moreover, the HupS-HaloTag residence time decreased from 0.236 s to 0.212 s during spore maturation in the absence of CobB1 (Figure 4B).

Our results indicate that HupS binding to DNA increases during *S. venezuelae* sporogenic development. Moreover, in the absence of CobB1, when HupS acetylation is enhanced, the fraction of immobile, DNA-bound protein molecules decreases, confirming that acetylation modulates HupS-DNA binding.

### Alterations in CobB1 levels affect *S. venezuelae* growth and decrease nucleoid compaction in spores

Considering the role of CobB1 in HupS deacetylation, the influence of HupS lysine modifications on its DNA binding, and the significance of HupS binding for DNA compaction, we expected to observe changes in nucleoid area in the *cobB1* deletion strain, similar to those described earlier in the *hupS* deficiency strain. Moreover, since *hupS* deletion resulted in decreased spore viability, we expected that *cobB1* deletion may also affect spore germination (18,19).

**Figure 5.**
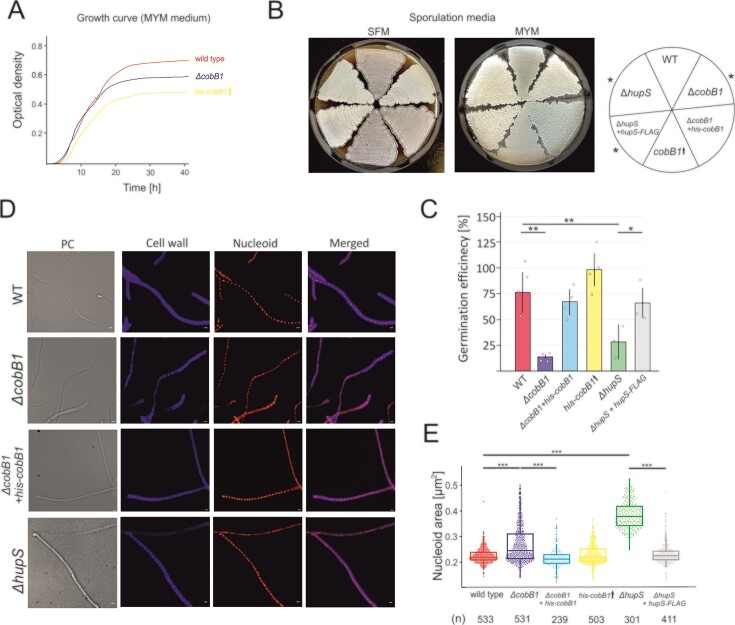
The impact of *cobB1* gene deletion on *S. venezuelae* growth, nucleoid compaction, and germination efficiency. (**A**) Analysis of the growth rates of *S. venezuelae* in liquid MYM. (**B**) The growth *S. venezuelae* strains on solid SFM and MYM media after 120 h. The aberrant colony pigmentation is marked with asterisk. (**C**) Quantification of the percent of colony forming units (CFU) in relation to total spore number (germination efficiency) of *S. venezuelae* strains cultivated on solid MYM medium. Each analysis was performed in 3–4 experimental replicates. Individual data points and standard deviations are marked in each bar. The statistical significance tested using an unpaired *t* test is marked (*P* value < 0.01, **). (**D**) Visualization of the nucleoid in *S. venezuelae* prespores. The cell wall was stained using wheat germ agglutinin (WGA) conjugated with AlexaFluor 350 (blue). Nucleoids were visualized using 7-amino-actinomycin D (7-AAD; red). The bright field, separate fluorescent channel, and overlay of fluorescence on brightfield images are shown as indicated. Scale bar: 1 μm. (**E**) Box plot analysis of the nucleoid area [μm^2^] in prespores of *S. venezuelae* stains growing for 22–24 on solid SFM medium. The boxplots show a median with the first and third quartile, while the lower and upper ‘whiskers’ extend to the value no further than 1.5 * QR (interquartile range) from the ‘hinge’. The statistical significance was determined with the Wilcoxon test. The significant differences (*P* value < 0.001. ***) are marked on the diagram. The presented data were collected in two separate experiments and combined. The number of analyzed spores (*n*) is shown below the box plot. All the presented *S. venezuelae* strains are listed below: wild-type (WT; red), *ΔcobB1* (JD01; violet), *ΔcobB1* complementation (*ΔcobB1*+ *his*-*cobB1*, JD11; blue), CobB1 overproduction (*his*-*cobB1↑*, JD04; yellow), *ΔhupS* (AKO200; green), and *ΔhupS* complementation (*ΔhupS* + *hupS*-FLAG, TM015; grey).

The analysis of the influence of *cobB1* deletion on the growth rate showed mild growth inhibition in comparison to the wild-type strain, detectable mostly during the stationary phase corresponding with sporogenic differentiation of *S. venez*uelae (Figure 5A). The analysis of the *ΔcobB1* strain growth on solid medium (rich MYM and sporulation-accelerating SFM medium) suggested aberrant colony pigmentation, similar to those observed for *hupS* deletion strain (Figure 5B), and corroborating the phenotype of the *hupS* deletion strain in *S. coelicolor* (19). Surprisingly, the colony pigmentation was not fully restored by the complementation of the *hupS* deletion strain with HupS-FLAG protein (Figure 5B). On the other hand, the effect of CobB1 overproduction (*his*-*cobB1*↑) was more pronounced. An increase in CobB1 levels lowered the growth rate in both liquid and solid medium (MYM) (Figures 5A and Supplementary Figure S8) but did not influence differentiation on minimal medium (MM) (Supplementary Figure S8) or colony pigmentation (Figure 5B).

The observed slowed culture growth and aberrant pigmentation of colonies of the *ΔcobB1* strain suggested the putative contribution of CobB1 deacetylase to spore development. To assess spore viability, we quantified the percentage of germinating spores (germination efficiency) for *ΔcobB1, cobB1* overexpression, and *ΔhupS* compared to wild-type and two complemented strains (*ΔcobB1 + his-cobB1* or *ΔhupS + hupS-flag*). The germination efficiency for the *ΔcobB1* strain (14% ± 2.2) was up to 5.4-fold less than that of the wild-type strain (76% ± 19.7) and comparable to that of the *hupS* deletion strain (29% ±12.5). When the *cobB1* gene under the control of the constitutive *ermE* promoter was delivered *in trans* in the *cobB1* deletion background (*ΔcobB1*+ *his*-*cobB1*, complementation strain), germination efficiency (67% ± 12.7) was comparable to that of the wild-type strain (Figure 5C) or *hupS* deletion strain complemented with *hupS*-FLAG under its native promoter (65% ± 13.4). On the other hand, the germination efficiency quantified for the *his*-*cobB1*↑ strain (98% ± 18.4) did not show any significant differences in comparison to the wild-type strain.

The observed similar impact of *cobB1* or *hupS* deletions on spore germination efficiency and the fact that HupS contributes to chromosome compaction prompted us to analyse nucleoid organisation in the absence of CobB1. We previously observed that *hupS* deletion significantly disturbed chromosome compaction, increasing the nucleoid area (18). Here, we analysed microscopically the late sporogenic hyphae of wild-type and *ΔcobB1* strains by measuring the nucleoid area. The microscopy observations did not indicate chromosome segregation defects in *ΔcobB1* strain compared to wild type strain (Figure 5D) corroborating the lack of segregation defects in *hupS* deletion strain observed previously in *S. venezuelae* (18). Moreover, and also similar to Δ*hupS* strain, *cobB1* gene deletion showed increased nucleoid areas and their higher variation compared to the wild-type strain (Figures 5D and E). However, the effect of *cobB1* deletion was weaker than that of *hupS* deletion, suggesting that *cobB1* deletion led to only mild nucleoid decompaction. The nucleoid area was restored by complementing the *cobB1* deletion with *in trans-*delivered *his*-*cobB1* gene on the integrative plasmid (*ΔcobB1*+ *his*-*cobB1* strain). This observation suggested that in the absence of CobB1 deacetylase, the overacetylated HupS had a lowered capacity for nucleoid compaction. Surprisingly, CobB1 overexpression (*his*-*cobB1*↑ strain) did not enhance detectable chromosome compaction (Figure 5E).

In summary, the analyses of *cobB1* deletion and overexpression in *S. venezuelae* showed that the altered CobB1 levels affect sporogenic development and spores maturation. Increased *cobB1* expression lead to growth retardation and influenced differentiation in rich medium. On the other hand, the absence of CobB1, which lead to increased acetylation of HupS, lowered nucleoid compaction in spores and affected spore maturation and their efficiency of germination similarly as *hupS* deletion.

## Discussion

While in bacteria, lysine acetylation was initially found to control acetyl-CoA synthetase (Acs) in *Salmonella enterica* (33,59), further studies showed a much broader impact of RLAs on cellular processes, including virulence (30), carbon and nitrogen metabolism (60,61), biofilm formation or motility (62), and stress response (63). Surprisingly, although many acetylome studies clearly indicated the high abundance of acetylated proteins in bacteria (64), including *Streptomyces*, the involvement of RLAs in the regulation of protein−DNA interactions and chromosome organisation has been scarcely described. However, the recent discovery of histone-like HU homologues being posttranslationally modified revealed novel molecular mechanisms that control bacterial chromosome organization and dynamics (28,30,53,65–67).

Here, we confirmed that the *S. venezuelae* HupS protein is subjected to lysine acetylation *in vivo*, corroborating the previously reported modifications of other HU homologues. Intriguingly, earlier LC−MS analyses of *S. griseus* and *S. coelicolor* acetylomes did not detect HupS acetylation *in vivo* (37,68). Although in our studies we confirmed with high accuracy acetylation of 2 (confirmed in two LC–MS experiments) up to 5 (confirmed at least in one experiment) lysine residues, 4 of them were positioned within, or close to, the N-terminal HU domain. Moreover, the complementary Western blotting analyses indicated acetylation within the LR domain (Supplementary Figure S2B). The lack of HupS in *Streptomyces* acetylomes determined by LC–MS in several studies, including ours, could be explained by the applied procedures in which the proteins were subjected to prolonged trypsin digestion (68). Due to the high abundance of lysine residues in HupS, extensive trypsin digestion could deliver very short peptides with low sequence uniqueness, leading to the discrimination of proteins that are rich in lysine repeats (69). However, in the acetylome of *S. griseus* investigated using anti-AcK antibodies, a protein with a molecular mass slightly below 30 kDa was detected to be extensively acetylated (68). Although not commented on by the authors in the manuscript, their findings resemble our acetylome studies on *S. venezuelae* (Figure 1).

In our studies, we restored the high HupS acetylation state *in vitro* by using AcP to achieve multiple lysine acetylation, covering also the lysine residues located in the LR domain. Due to LC–MS limitations, we were not able to quantify the number of *in vivo* acetylated lysine residues in HupS. However, based on the complementary Western blotting analysis, we hypothesize that HupS is also a multi-lysine-acetylated protein, similar to the *M. tuberculosis* HupB homologue, in which the number of identified acetylated lysine residues varies from 8 residues for endogenous MtHupB (28) up to 31 if MtHupB was acetylated *in vitro* by a GNAT-like acetyltransferase named Eis (30). Overproduction of the Eis protein in *M. smegmatis* led to HupB hyperacetylation *in vivo* (28). Although Eis can serve as a potent HupB acetylase (with low and/or unknown sequence specificity), nonspecific HupB acetylation *in vivo* by AcCoA or AcP cannot be excluded. However, the cellular mechanism of HupS acetylation is not known. Since *Streptomyces* genomes encode up to 72 putative acetyltransferases (70), the identification of any HupS-dedicated acetyltransferase is highly challenging.

Given that lysine-rich LR domains are identified predominantly in DNA-binding proteins (27) and taking into account that histone tail acetylation affects their interaction with DNA (28), we expected that the identified HupS acetylation would affect DNA affinity. According to our prediction, the acetylated recombinant His-HupS showed an approximately 2-fold lower affinity for single- and double-stranded DNA in comparison to the nonacetylated protein. Interestingly, acetylation of K86 residue was shown to modify *E. coli* HUβ binding to different DNA substrates, promoting its binding to long DNA fragments and decreasing the affinity for nicked or gapped DNA (66). Although we detected that HupS shows a preference for supercoiled or single-stranded DNA, corroborating earlier observations in *E. coli* (71), the decreased DNA affinity resulting from HupS nonspecific acetylation was comparable for all tested DNA substrates. Moreover, we noticed that His-HupS acetylation did not influence homodimer formation, which could explain the decreased protein−DNA interactions observed for *E. coli* HUβ (66).

Since the major role of HupS is DNA compaction in spores, HupS levels increase strongly prior to sporulation (18,19), but the exact role of HupS acetylation during *Streptomyces* development remains unclear. Surprisingly, we found that HupS is constantly acetylated during *S. venezuelae* development or that acetylation levels even slightly increase during spore maturation when hyphal growth is arrested and following the gradual increase in protein acetylation in *S. venezuelae*. This observation can be explained by the observation that in general, protein acetylation is strongly induced during sporogenic development in *S. griseus* (68) and *S. venezuelae* (this study). The source of protein acetylation during sporulation is unclear. However, recent studies have shown that AcP is able to acetylate *S. coelicolor* proteins nonspecifically *in vitro* (72) and that AcP levels increase during the stationary phase, which in *E. coli* leads to the accumulation of acetylated proteins while growth and cell division are arrested (73). Most bacterial species generate AcP by transferring an acetyl group from AcCoA into inorganic phosphate (Pta-dependent pathway) or by acetate fermentation (AckA-dependent pathway), and both *pta* and *ackA* genes are present in *Streptomyces* genomes. Thus, constant HupS acetylation might be a natural consequence of LR domain exposure to AcP. Lysine susceptibility to nonenzymatic AcP-dependent acetylation is strongly correlated with lysine exposure to the solvent and/or the presence of particular amino acid residues. The proximity of specific amino acids (Thr, Ser, Asn, Gln) promotes AcP coordination (74). Thus, the long and intrinsically disordered C-terminal domain of *Streptomyces* HupS or mycobacterial HupB, in which KK doublets are separated by several serine or threonine residues, could serve as a preferable target for AcP-dependent multiple, nonenzymatic lysine acetylation *in vivo*. Given the increase CobB1 levels during sporulation, we speculate that its major role is presumably preventing nonspecific overacetylation of HupS. Our studies showed that CobB1 is responsible for HupS deacetylation *in vitro* promoting HupS-DNA interactions. Our observation that acetylated HupS binds DNA with lower affinity corroborates the lowered fraction of DNA-bound protein molecules in *S. venezuelae* spores as detected in the *cobB1* deletion strain. However, since the CobB1 deacetylase activity *in vitro* was low (also shown by other groups (36,37)), we hypothesize that CobB1 activity *in vivo* could be stimulated by unknown cellular factors including PTMs and/or interactions with other macromolecules or cofactors.

The observation that *cobB1* deletion has no effect on *S. venezuelae* vegetative growth in all tested media, including rich and minimal media, solid and liquid, prompted us to hypothesize that CobB1 activity is particularly important when *Streptomyces* growth is arrested and the colony differentiates into spores. As assumed, the *cobB1* deletion strain had aberrant colony pigmentation similar to those observed in the *hupS* deletion strain, suggesting the involvement of HupS acetylation/deacetylation state controlled by CobB1 in spore maturation. Moreover, based on the observation that HupS-FLAG does not fully complement *hupS* deletion phenotype, we speculate that either expression of *hupS-flag* from different chromosomal *locus*, or FLAG-tag may have impact on HupS activity *in vivo* i.e. by affecting protein (including CobB1) accessibility to the LR domain of HupS-FLAG. The particular importance of CobB1 during sporogenic development is also supported by the observation that in *S. coelicolor*, the CobB1 homologue specifically deacetylates ParB, a segregation protein that assists chromosome distribution to prespores (36). In *S. coelicolor*, the lack of *cobB1* gene leads to chromosome segregation defects (22.5%) comparable with the *parB* deletion (17,4%) (53,75,76). However, the lysine residue acetylated in *S. coelicolor* ParB (53) is not conserved in *S. venezuelae*, in which it is substituted with positively charged, but acetylation insensitive arginine. These observations suggest, that the role of CobB1 during sporogenic development is not conserved and may differ between particular *Streptomyces* species.

It should be noted, when CobB1 was overproduced (as a second copy under the constitutive *ermE* promoter), the impact on vegetative growth was stronger and less clear. We observed strong growth and development inhibition of the CobB1-overproducing strain, but only in a rich medium. This suggests that the overproduction of CobB1 protein can have a pleiotropic effect on cell functioning, which is highly dependent on nutrient availability. Intriguingly, the observation that CobB1 overproduction does not affect the number of germinating spores but decreases the growth rate on rich medium leads us to speculate that CobB1 activity may be also required during germination or, at least, that increased deacetylase activity does not disturb the process. On the other hand, during vegetative growth, when the *cobB1* gene is typically downregulated, *cobB1* overexpression may lead to the deregulation of cell metabolism. In *S. coelicolor*, among 601 proteins identified as acetylated, 36.5% are involved in essential cell metabolism, including glycolysis, the tricarboxylic acid (TCA) cycle, and energy metabolism. Thus, the deregulation of the key metabolic pathways, normally controlled by the RLAs, in the presence of high deacetylase activity of CobB1 could explain the growth inhibition of the *cobB1* mutant.

In summary, our studies showed that the HupS protein, a close HU homologue, is acetylated in *S. venezuelae*, decreasing its capacity for DNA interactions *in vitro* and *in vivo*. Since we detected that protein acetylation increases during *S. venezuelae* sporogenic differentiation, HupS overacetylation could negatively affect chromosome compaction in spores, thus requiring CobB1 deacetylase activity.

## Supplementary Material

gkae418_Supplemental_Files

## Data Availability

The RNA-Seq data extracted in this study (shown in Figure 3A and Supplementary Figure S4C) are available in the Array Express database (EMBL-EBI) under accession number **E-MTAB-13607** (46) and **E-MTAB-13911** (47), respectively. The mass spectrometry proteomics data have been deposited to the deposited to the ProteomeXChange Consortium via the PRIDE partner repository (77). The LC-MS data shown in Figure 1A and Supplementary Figure S2A data base under accession number **PXD050776**. The LC-MS data shown in Figure 2C and Supplementary Figure S2C were deposited under accession number **PXD048203**.

## Notes

Present address: Justyna Szymczak, Department of Cell Biology, University of Oklahoma, Oklahoma City, OK 73104, USA.

## References

[1] Zimmerman S.B. Shape and compaction of Escherichia coli nucleoids. J. Struct. Biol. 2006; 156:255–261. 16697220 10.1016/j.jsb.2006.03.022

[2] Murphy L.D. , Zimmerman S.B. Condensation and cohesion of lambda DNA in cell extracts and other media: implications for the structure and function of DNA in prokaryotes. Biophys. Chem. 1995; 57:71–92. 8534838 10.1016/0301-4622(95)00047-2

[3] Dorman C.J. , Dorman M.J. DNA supercoiling is a fundamental regulatory principle in the control of bacterial gene expression. Biophys. Rev. 2016; 8:209–220. 28510224 10.1007/s12551-016-0205-y PMC5425793

[4] Badrinarayanan A. , Le T.B.K., Laub M.T. Bacterial chromosome organization and segregation. Annu. Rev. Cell Dev. Biol. 2015; 31:171–199. 26566111 10.1146/annurev-cellbio-100814-125211 PMC4706359

[5] Szafran M.J. , Jakimowicz D., Elliot M.A. Compaction and control—the role of chromosome-organizing proteins in streptomyces. FEMS Microbiol. Rev. 2020; 44:725–739. 32658291 10.1093/femsre/fuaa028 PMC7685783

[6] Amemiya H.M. , Schroeder J., Freddolino P.L. Nucleoid-associated proteins shape chromatin structure and transcriptional regulation across the bacterial kingdom. Transcription. 2021; 12:182–218. 34499567 10.1080/21541264.2021.1973865 PMC8632127

[7] Norris V. , Kayser C., Muskhelishvili G., Konto-Ghiorghi Y. The roles of nucleoid-associated proteins and topoisomerases in chromosome structure, strand segregation and the generation of phenotypic heterogeneity in bacteria. FEMS Microbiol. Rev. 2022; 47:fuac049. 10.1093/femsre/fuac049 36549664

[8] Dame R.T. , Kalmykowa O.J., Grainger D.C. Chromosomal macrodomains and associated proteins: implications for DNA organization and replication in gram negative bacteria. PLoS Genet. 2011; 7:e1002123. 21698131 10.1371/journal.pgen.1002123 PMC3116907

[9] Hardy C.D. , Cozzarelli N.R. A genetic selection for supercoiling mutants of Escherichia coli reveals proteins implicated in chromosome structure. Mol. Microbiol. 2005; 57:1636–1652. 16135230 10.1111/j.1365-2958.2005.04799.x

[10] Browning D.F. , Grainger D.C., Busby S.J. Effects of nucleoid-associated proteins on bacterial chromosome structure and gene expression. Curr. Opin. Microbiol. 2010; 13:773–780. 20951079 10.1016/j.mib.2010.09.013

[11] Grove A. Functional evolution of bacterial histone-like HU proteins. Curr. Iss. Mol. Biol. 2011; 13:1–12. 20484776

[12] Rouvière-Yaniv J. , Gros F. Characterization of a novel, low-molecular-weight DNA-binding protein from Escherichia coli. Proc. Natl. Acad. Sci. U.S.A. 1975; 72:3428–3432. 1103148 10.1073/pnas.72.9.3428 PMC433007

[13] Ali Azam T. , Iwata A., Nishimura A., Ueda S., Ishihama A Growth phase-dependent variation in protein composition of the Escherichia coli nucleoid. J. Bacteriol. 1999; 181:6361–6370. 10515926 10.1128/jb.181.20.6361-6370.1999 PMC103771

[14] Flärdh K. , Buttner M.J. Streptomyces morphogenetics: dissecting differentiation in a filamentous bacterium. Nat. Rev. Micro. 2009; 7:36–49. 10.1038/nrmicro1968 19079351

[15] McCormick J.R. , Flärdh K. Signals and regulators that govern streptomyces development. FEMS Microbiol. Rev. 2012; 36:206–231. 22092088 10.1111/j.1574-6976.2011.00317.x PMC3285474

[16] Bush M.J. , Bibb M.J., Chandra G., Findlay K.C., Buttner M.J. Genes required for aerial growth, cell division, and chromosome segregation are targets of WhiA before sporulation in streptomyces venezuelae. mBio. 2013; 4:e00684-13. 24065632 10.1128/mBio.00684-13 PMC3781837

[17] Donczew M. , Mackiewicz P., Wróbel A., Flärdh K., Zakrzewska-Czerwińska J., Jakimowicz D. ParA and ParB coordinate chromosome segregation with cell elongation and division during streptomyces sporulation. Open Biol. 2016; 6:150263. 27248800 10.1098/rsob.150263 PMC4852455

[18] Szafran M.J. , Małecki T., Strzałka A., Pawlikiewicz K., Duława J., Zarek A., Kois-Ostrowska A., Findlay K.C., Le T.B.K., Jakimowicz D. Spatial rearrangement of the Streptomyces venezuelae linear chromosome during sporogenic development. Nat. Commun. 2021; 12:5222. 34471115 10.1038/s41467-021-25461-2 PMC8410768

[19] Salerno P. , Larsson J., Bucca G., Laing E., Smith C.P., Flärdh K. One of the two genes encoding nucleoid-associated HU proteins in Streptomyces coelicolor is developmentally regulated and specifically involved in spore maturation. J. Bacteriol. 2009; 191:6489–6500. 19717607 10.1128/JB.00709-09 PMC2795297

[20] Mukherjee A. , DiMario P.J., Grove A. Mycobacterium smegmatis histone-like protein hlp is nucleoid associated. FEMS Microbiol. Lett. 2009; 291:232–240. 19146577 10.1111/j.1574-6968.2008.01458.x

[21] Cohavy O. , Harth G., Horwitz M., Eggena M., Landers C., Sutton C., Targan S.R., Braun J. Identification of a novel mycobacterial histone H1 homologue (HupB) as an antigenic target of pANCA monoclonal antibody and serum immunoglobulin A from patients with Crohn's disease. Infect. Immun. 1999; 67:6510–6517. 10569769 10.1128/iai.67.12.6510-6517.1999 PMC97061

[22] Hołówka J. , Trojanowski D., Ginda K., Wojtaś B., Gielniewski B., Jakimowicz D., Zakrzewska-Czerwińska J. HupB is a bacterial nucleoid-associated protein with an indispensable eukaryotic-like tail. mBio. 2017; 8:e01272-17. 29114022 10.1128/mBio.01272-17 PMC5676037

[23] Singh N. , Sharma N., Singh P., Pandey M., Ilyas M., Sisodiya L., Choudhury T., Gosain T.P., Singh R., Atmakuri K. HupB, a nucleoid-associated protein, is critical for survival of mycobacterium tuberculosis under host-mediated stresses and for enhanced tolerance to key first-line antibiotics. Front. Microbiol. 2022; 13:937970. 36071978 10.3389/fmicb.2022.937970 PMC9441915

[24] Katsube T. , Matsumoto S., Takatsuka M., Okuyama M., Ozeki Y., Naito M., Nishiuchi Y., Fujiwara N., Yoshimura M., Tsuboi T. et al. Control of cell wall assembly by a histone-like protein in mycobacteria. J. Bacteriol. 2007; 189:8241–8249. 17873049 10.1128/JB.00550-07 PMC2168677

[25] Whiteford D.C. , Klingelhoets J.J., Bambenek M.H., Dahl J.L. Deletion of the histone-like protein (Hlp) from Mycobacterium smegmatis results in increased sensitivity to UV exposure, freezing and isoniazid. Microbiology (Reading). 2011; 157:327–335. 20966096 10.1099/mic.0.045518-0

[26] Sharadamma N. , Khan K., Kumar S., Patil K.N., Hasnain S.E., Muniyappa K. Synergy between the N-terminal and C-terminal domains of Mycobacterium tuberculosis HupB is essential for high-affinity binding, DNA supercoiling and inhibition of RecA-promoted strand exchange. FEBS J. 2011; 278:3447–3462. 21787377 10.1111/j.1742-4658.2011.08267.x

[27] Strzalka A. , Szafran M.J., Strick T., Jakimowicz D. C-terminal lysine repeats in Streptomyces topoisomerase I stabilize the enzyme-DNA complex and confer high enzyme processivity. Nucleic Acids Res. 2017; 45:11908–11924. 28981718 10.1093/nar/gkx827 PMC5714199

[28] Ghosh S. , Padmanabhan B., Anand C., Nagaraja V. Lysine acetylation of the mycobacterium tuberculosis HU protein modulates its DNA binding and genome organization. Mol. Microbiol. 2016; 100:577–588. 26817737 10.1111/mmi.13339

[29] Yaseen I. , Choudhury M., Sritharan M., Khosla S. Histone methyltransferase SUV39H1 participates in host defense by methylating mycobacterial histone-like protein HupB. EMBO J. 2018; 37:183–200. 29170282 10.15252/embj.201796918 PMC5771397

[30] Green K.D. , Biswas T., Pang A.H., Willby M.J., Reed M.S., Stuchlik O., Pohl J., Posey J.E., Tsodikov O.V., Garneau-Tsodikova S. Acetylation by Eis and deacetylation by Rv1151c of mycobacterium tuberculosis HupB: biochemical and structural insight. Biochemistry. 2018; 57:781–790. 29345920 10.1021/acs.biochem.7b01089 PMC5971062

[31] Sakatos A. , Babunovic G.H., Chase M.R., Dills A., Leszyk J., Rosebrock T., Bryson B., Fortune S.M. Posttranslational modification of a histone-like protein regulates phenotypic resistance to isoniazid in mycobacteria. Sci. Adv. 2018; 4:eaao1478. 29732401 10.1126/sciadv.aao1478 PMC5931751

[32] VanDrisse C.M. , Escalante-Semerena J.C. Protein acetylation in bacteria. Annu. Rev. Microbiol. 2019; 73:111–132. 31091420 10.1146/annurev-micro-020518-115526 PMC6736716

[33] Starai V.J. , Escalante-Semerena J.C. Identification of the protein acetyltransferase (Pat) enzyme that acetylates acetyl-CoA synthetase in Salmonella enterica. J. Mol. Biol. 2004; 340:1005–1012. 15236963 10.1016/j.jmb.2004.05.010

[34] Hoff K.G. , Avalos J.L., Sens K., Wolberger C. Insights into the sirtuin mechanism from ternary complexes containing NAD+ and acetylated peptide. Structure. 2006; 14:1231–1240. 16905097 10.1016/j.str.2006.06.006

[35] Carabetta V.J. , Cristea I.M. Regulation, function, and detection of protein acetylation in bacteria. J. Bacteriol. 2017; 199:e00107–e17. 28439035 10.1128/JB.00107-17 PMC5527388

[36] Mikulik K. , Felsberg J., Kudrnáčová E., Bezoušková S., Setinová D., Stodůlková E., Zídková J., Zídek V. CobB1 deacetylase activity in Streptomyces coelicolor. Biochem. Cell. Biol. 2012; 90:179–187. 22300453 10.1139/o11-086

[37] Yang Y. , Zhang H., Guo Z., Zou S., Long F., Wu J., Li P., Zhao G.-P., Zhao W. Global insights into lysine acylomes reveal crosstalk between lysine acetylation and succinylation in streptomyces coelicolor metabolic pathways. Mol. Cell. Proteomics. 2021; 20:100148. 34530157 10.1016/j.mcpro.2021.100148 PMC8498004

[38] Liao G. , Xie L., Li X., Cheng Z., Xie J. Unexpected extensive lysine acetylation in the trump-card antibiotic producer Streptomyces roseosporus revealed by proteome-wide profiling. J. Proteomics. 2014; 106:260–269. 24768905 10.1016/j.jprot.2014.04.017

[39] Sambrook J. , Russell D. Molecular Cloning: A Laboratory Manual. 2001; 1, 2 and 3:3rd edn. NY Cold Spring Harbor Laboratory Press.

[40] Kieser T. , Bibb M.J., Buttner M., Chater K., Hopwood D.A. Practical streptomyces genetics The John Innes Foundation. 2000;

[41] Szafran M. , Skut P., Ditkowski B., Ginda K., Chandra G., Zakrzewska-Czerwińska J., Jakimowicz D. Topoisomerase I (TopA) is recruited to ParB complexes and is required for proper chromosome organization during streptomyces coelicolor sporulation. J. Bacteriol. 2013; 195:4445–4455. 23913317 10.1128/JB.00798-13 PMC3807468

[42] Rappsilber J. , Ishihama Y., Mann M. Stop and go extraction tips for matrix-assisted laser desorption/ionization, nanoelectrospray, and LC/MS sample pretreatment in proteomics. Anal. Chem. 2003; 75:663–670. 12585499 10.1021/ac026117i

[43] Distler U. , Kuharev J., Navarro P., Tenzer S. Label-free quantification in ion mobility-enhanced data-independent acquisition proteomics. Nat. Protoc. 2016; 11:795–812. 27010757 10.1038/nprot.2016.042

[44] Zee B.M. , Garcia B.A. Discovery of lysine post-translational modifications through mass spectrometric detection. Essays Biochem. 2012; 52:147–163. 22708569 10.1042/bse0520147 PMC4113086

[45] Yang L. , Tu S., Ren C., Bulloch E.M.M., Liao C.-L., Tsai M.-D., Freitas M.A. Unambiguous determination of isobaric histone modifications by reversed-phase retention time and high-mass accuracy. Anal. Biochem. 2010; 396:13–22. 19699711 10.1016/j.ab.2009.08.027 PMC2787863

[46] Duława-Kobeluszczyk J. , Mikołajczyk J., Strzałka A., Jakimowicz D., Szafran M. Global transcript levels during growth of Streptomyces venezuelae wild-type strain. 2023; BioStudies, E-MTAB-13607.

[47] Duława-Kobeluszczyk J. , Mikołajczyk J., Strzałka A., Jakimowicz D., Szafran M. The impact of cobB1 or cobB2 gene deletions on global transcript levels in Streptomyces venezuelae. 2024; BioStudies, E-MTAB-13911.

[48] Tinevez J.-Y. , Perry N., Schindelin J., Hoopes G.M., Reynolds G.D., Laplantine E., Bednarek S.Y., Shorte S.L., Eliceiri K.W. TrackMate: an open and extensible platform for single-particle tracking. Methods. 2017; 115:80–90. 27713081 10.1016/j.ymeth.2016.09.016

[49] Rösch T.C. , Oviedo-Bocanegra L.M., Fritz G., Graumann P.L. SMTracker: a tool for quantitative analysis, exploration and visualization of single-molecule tracking data reveals highly dynamic binding of B. subtilis global repressor AbrB throughout the genome. Sci. Rep. 2018; 8:15747. 30356068 10.1038/s41598-018-33842-9 PMC6200787

[50] Paintdakhi A. , Parry B., Campos M., Irnov I., Elf J., Surovtsev I., Jacobs-Wagner C. Oufti: an integrated software package for high-accuracy, high-throughput quantitative microscopy analysis. Mol. Microbiol. 2016; 99:767–777. 26538279 10.1111/mmi.13264 PMC4752901

[51] Suka N. , Suka Y., Carmen A.A., Wu J., Grunstein M. Highly specific antibodies determine histone acetylation site usage in yeast heterochromatin and euchromatin. Mol. Cell. 2001; 8:473–479. 11545749 10.1016/s1097-2765(01)00301-x

[52] Weinert B.T. , Iesmantavicius V., Wagner S.A., Schölz C., Gummesson B., Beli P., Nyström T., Choudhary C. Acetyl-phosphate is a critical determinant of lysine acetylation in E. coli. Mol. Cell. 2013; 51:265–272. 23830618 10.1016/j.molcel.2013.06.003

[53] Li P. , Zhang H., Zhao G.-P., Zhao W. Deacetylation enhances ParB-DNA interactions affecting chromosome segregation in Streptomyces coelicolor. Nucleic Acids Res. 2020; 48:4902–4914. 32313947 10.1093/nar/gkaa245 PMC7229854

[54] Claret L. , Rouviere-Yaniv J. Variation in HU composition during growth of Escherichia coli: the heterodimer is required for long term survival. J. Mol. Biol. 1997; 273:93–104. 9367749 10.1006/jmbi.1997.1310

[55] Kapanidis A.N. , Uphoff S., Stracy M. Understanding protein mobility in bacteria by tracking single molecules. J. Mol. Biol. 2018; 430:4443–4455. 29753778 10.1016/j.jmb.2018.05.002 PMC6198114

[56] El Sayyed H. , Pambos O.J., Stracy M., Gottesman M.E., Kapanidis A.N. Single-molecule tracking reveals the functional allocation, in vivo interactions, and spatial organization of universal transcription factor NusG. Mol. Cell. 2024; 84:926–937. 38387461 10.1016/j.molcel.2024.01.025 PMC7618293

[57] Garza de Leon F. , Sellars L., Stracy M., Busby S.J.W., Kapanidis A.N. Tracking low-copy transcription factors in living bacteria: the case of the lac repressor. Biophys. J. 2017; 112:1316–1327. 28402875 10.1016/j.bpj.2017.02.028 PMC5390046

[58] Strzałka A. , Kois-Ostrowska A., Kędra M., Łebkowski T., Bieniarz G., Szafran M.J., Jakimowicz D. Enhanced binding of an HU homologue under increased DNA supercoiling preserves chromosome organisation and sustains streptomyces hyphal growth. Nucleic Acids Res. 2022; 50:12202–12216. 36420903 10.1093/nar/gkac1093 PMC9756944

[59] Starai V.J. , Celic I., Cole R.N., Boeke J.D., Escalante-Semerena J.C. Sir2-dependent activation of acetyl-CoA synthetase by deacetylation of active lysine. Science. 2002; 298:2390–2392. 12493915 10.1126/science.1077650

[60] You D. , Wang M.-M., Ye B.-C. Acetyl-CoA synthetases of saccharopolyspora erythraea are regulated by the nitrogen response regulator GlnR at both transcriptional and post-translational levels. Mol. Microbiol. 2017; 103:845–859. 27987242 10.1111/mmi.13595

[61] Amin R. , Franz-Wachtel M., Tiffert Y., Heberer M., Meky M., Ahmed Y., Matthews A., Krysenko S., Jakobi M., Hinder M. et al. Post-translational serine/threonine phosphorylation and lysine acetylation: a novel regulatory aspect of the Global nitrogen response regulator GlnR in S. coelicolor M145. Front. Mol. Biosci. 2016; 3:38. 27556027 10.3389/fmolb.2016.00038 PMC4977719

[62] Reverdy A. , Chen Y., Hunter E., Gozzi K., Chai Y. Protein lysine acetylation plays a regulatory role in Bacillus subtilis multicellularity. PLoS One. 2018; 13:e0204687. 30265683 10.1371/journal.pone.0204687 PMC6161898

[63] Ren J. , Sang Y., Ni J., Tao J., Lu J., Zhao M., Yao Y.-F. Acetylation regulates survival of Salmonella enterica serovar typhimurium under acid stress. Appl. Environ. Microb. 2015; 81:5675–5682. 10.1128/AEM.01009-15 PMC4551269 26070677

[64] Christensen D.G. , Baumgartner J.T., Xie X., Jew K.M., Basisty N., Schilling B., Kuhn M.L., Wolfe A.J. Mechanisms, detection, and relevance of protein acetylation in prokaryotes. mBio. 2019; 10:e02708-18. 30967470 10.1128/mBio.02708-18 PMC6456759

[65] Zhou Q. , Zhou Y.N., Jin D.J., Tse-Dinh Y.-C. Deacetylation of topoisomerase I is an important physiological function of E. coli CobB. Nucleic Acids Res. 2017; 45:5349–5358. 28398568 10.1093/nar/gkx250 PMC5605244

[66] Barlow V.L. , Tsai Y.-H. Acetylation at lysine 86 of Escherichia coli huβ modulates the DNA-binding capability of the protein. Front. Microbiol. 2021; 12:809030. 35185833 10.3389/fmicb.2021.809030 PMC8854993

[67] Liao J.-H. , Tsai C.-H., Patel S.G., Yang J.-T., Tu I.-F., Lo Cicero M., Lipka-Lloyd M., Wu W.-L., Shen W.-J., Ho M.-R. et al. Acetylome of Acinetobacter baumannii SK17 reveals a highly-conserved modification of histone-like protein HU. Front Mol. Biosci. 2017; 4:77. 29230394 10.3389/fmolb.2017.00077 PMC5711770

[68] Ishigaki Y. , Akanuma G., Yoshida M., Horinouchi S., Kosono S., Ohnishi Y. Protein acetylation involved in streptomycin biosynthesis in Streptomyces griseus. J. Proteomics. 2017; 155:63–72. 28034645 10.1016/j.jprot.2016.12.006

[69] Yoshida Y. , Nishiyama A., Suameitria Dewi D.N.S., Yamazaki T., Yokoyama A., Kobayashi D., Kondo H., Ozeki Y., Matsumoto S. Limited proteolysis of mycobacterial DNA-binding protein 1 with an extended, lysine-rich, intrinsically disordered region to unveil posttranslational modifications. Biochem. Biophys. Res. Commun. 2023; 681:111–119. 37774568 10.1016/j.bbrc.2023.09.028

[70] Lu Y.-X. , Liu X.-X., Liu W.-B., Ye B.-C. Identification and characterization of two types of amino acid-regulated acetyltransferases in actinobacteria. Biosci. Rep. 2017; 37:BSR20170157. 28539332 10.1042/BSR20170157 PMC6434083

[71] Kamashev D. , Rouviere-Yaniv J. The histone-like protein HU binds specifically to DNA recombination and repair intermediates. EMBO J. 2000; 19:6527–6535. 11101525 10.1093/emboj/19.23.6527 PMC305869

[72] Takahashi-Íñiguez T. , Flores M.E. Acetyl phosphate acetylates proteins of streptomyces coelicolor M-145. Appl. Biochem. Microbiol. 2023; 59:450–455.

[73] Schilling B. , Christensen D., Davis R., Sahu A.K., Hu L.I., Walker-Peddakotla A., Sorensen D.J., Zemaitaitis B., Gibson B.W., Wolfe A.J. Protein acetylation dynamics in response to carbon overflow in Escherichia coli. Mol. Microbiol. 2015; 98:847–863. 26264774 10.1111/mmi.13161 PMC4715485

[74] Wang M.-M. , You D., Ye B.-C. Site-specific and kinetic characterization of enzymatic and nonenzymatic protein acetylation in bacteria. Sci. Rep. 2017; 7:14790. 29093482 10.1038/s41598-017-13897-w PMC5665961

[75] Kim H.J. , Calcutt M.J., Schmidt F.J., Chater K.F. Partitioning of the linear chromosome during sporulation of Streptomyces coelicolor A3(2) involves an oriC-linked parAB locus. J. Bacteriol. 2000; 182:1313–1320. 10671452 10.1128/jb.182.5.1313-1320.2000 PMC94417

[76] Jakimowicz D. , Chater K., Zakrzewska-Czerwínska J. The ParB protein of Streptomyces coelicolor A3(2) recognizes a cluster of parS sequences within the origin-proximal region of the linear chromosome. Mol. Microbiol. 2002; 45:1365–1377. 12207703 10.1046/j.1365-2958.2002.03102.x

[77] Perez-Riverol Y. , Bai J., Bandla C., García-Seisdedos D., Hewapathirana S., Kamatchinathan S., Kundu D.J., Prakash A., Frericks-Zipper A., Eisenacher M. et al. The PRIDE database resources in 2022: a hub for mass spectrometry-based proteomics evidences. Nucleic Acids Res. 2022; 50:D543–D552. 34723319 10.1093/nar/gkab1038 PMC8728295

