# Supplementary material for: The activity of CobB1 protein deacetylase contributes to nucleoid compaction in Streptomyces venezuelae spores by increasing HupS affinity for DNA
Source: Nucleic Acids Res. 2024 May 23;52(12):7112–28. doi: 10.1093/nar/gkae418 (PMC11229371; doi:10.1093/nar/gkae418)
Supplement: gkae418_Supplemental_Files [file gkae418_supplemental_files.zip › Supplementary Materials and Methods_REVISED.docx]

**Supplementary Materials and Methods**

**Plasmids and strains constructions**

To construct the *S. venezuelae* strain with the *cobB1* (*vnz_03080*) gene deletion, the CRISPR-Cas9 method and pCRISPomyces–2 plasmid were used (1). First, the 24-nucleotide long protospacers containing the PAM sequence (oligo_*cobB1*_RV or oligo_*cobB2*_RV) as well as the complementary oligonucleotide (oligo_*cobB1*_FW or oligo_*cobB2*_FW) were phosphorylated, annealed, and used as a *cobB1*_sgRNA and *cobB2*_sgRNA sequences, respectively, for Golden Gate assembly (2) into *BbsI*-digested pCRISPomyces-2 plasmid. The reaction yielded pCRISPomyces-2_*cobB1* and pCRISPomyces-2_*cobB2* plasmids, which were verified by Sanger sequencing. Next, the obtained pCRISPomyces-2_*cobB1* and pCRISPomyces-2_*cobB2* were used to clone two DNA fragments, each 1000 bp upstream (FR1) or downstream (FR2) regions of the *cobB1* or *cobB2* genes, respectively. These fragments were amplified using oligonucleotides *cobB1*_FR1_FW and *cobB1*_FR1_RV (or *cobB2*_FR1_FW and *cobB2*_FR1_RV) as well as *cobB1*_FR2_FW and *cobB1*_FR2_RV (or *cobB2*_FR1_FW and *cobB2*_FR1_RV) for FR1 and FR2 DNA fragments flanking *cobB1* or *cobB2* genes, respectively, and carried 20-nucleotide long overlapping DNA sequences. The FR1 and FR2 fragments were purified from agarose gel and ligated with *XbaI*-digested pCRISPomyces-2_*cobB1* plasmid using the SLIC method (3), yielding pCRISPomyces-2_*cobB1*-MH and pCRISPomyces-2_*cobB2*-MH plasmids (verified by sequencing using Seq_oligo_fwd oligonucleotide). The obtained plasmids were next transformed into a chemically competent *E. coli* ET12567/pUZ8002 strain which was used for conjugation with the wild-type *S. venezuelae* strain. The apramycin-resistant *S. venezuelae* exconjugates were selected on the R2 medium (4) not supplemented with sucrose and after a few days restreaked on fresh MYM agar plates, both media supplemented with apramycin. The apramycin-resistant clones were then transferred to a liquid MYM medium and cultivated for 24 hours at 30°C with 180 rpm shaking. The pCRISPomyces-plasmid clearance was accomplished with overnight cultivation of the selected clones at 39 °C, followed by replica plating on selective (apramycin) and non-selective (without antibiotics) MYM plates to confirm the restoration of apramycin sensitivity, and yielding JD01 strain (*∆cobB1*) or JD07 (*∆cobB2*). The chromosomal DNA was isolated using a Genomic Mini AX Streptomyces kit (A&A Biotechnology, Poland), and the introduced mutation was verified by PCR reaction using Del_*cobB1*_FW and Del_*cobB1*_RV (for *cobB1* deletion) or Del_*cobB1*_FW and Del_*cobB1*_RV (for *cobB2* deletion) oligonucleotides. To construct double *∆cobB1/∆cobB2* deletion strain (JD08), the JD01 strain was conjugated with the *E. coli* ET12567/pUZ8002 carrying pCRISPomyces-2_*cobB2*-MH plasmid and followed the selection procedure described above.

To construct the *S. venezuelae* strains producing HupS-HaloTag, the pIJ10770-*hupS-halotag* plasmid, containing *hupS-halotag* gene under the control of the native *hupS* promoter was delivered *in* *trans* as the second copy of the *hupS* gene. To obtain pIJ10770-*hupS-halotag* plasmid, first the *halotag* sequence was amplified on the template of PFC 20K HaloTag T7 SP6 Flexi Vector (5) using primers pss170-linker-HT(C)_fw and pss170-HT(C)_rv, and ligated with *XhoI*/*XmaI*-digested pIJ10770 plasmid (Hyg^R^) )(6). In parallel, *hupS* gene with its native promoter was amplified using primers *hupS*-tomcherry-2-FW and *hupS*-toHT_RV on the template of pIJ10770-*hupS-mcherry* plasmid (the plasmid constructed earlier by amplifying *hupS* gene with in upstream region using *hupS*_tomcherry_FW and *hupS*_tomcherry_RV oligonucleotides, and ligated with *NdeI*- and *XhoI*-digested pIJ10770 plasmid). Next, the PCR product was cloned into pGEM-T-Easy vector (Promega, US) using TA-cloning yielding pGEM-*hupS* plasmid. Then, pGEM-*hupS* was digested with *NdeI* and *XmaI* restriction enzymes, and the 902 pz DNA fragment encoding *hupS* gene with its native promoter was cloned into *NdeI/XmaI-*digested pIJ10770-*halotag* plasmid. The obtained pIJ10770-*hupS*-*halotag* plasmid was verified by sequencing and subsequently delivered by conjugation, as described earlier, to *S. venezuelae* wild-type and JD01 strains, yielding AZ01 and JD14 strains, respectively. The exconjugants were selected using hygromycin and verified by colony PCR using primers pSS_seq_FW and pSS_seq_RV.

To construct the *S. venezuelae* strains producing CobB1 protein with N-terminally fused HisTag (His-CobB1) we used pIJ10770_*ermE*_*halotag* plasmid. First, a fragment encoding *ermE*p promoter was amplified using SLIC_*ermE*_FW and SLIC_*ermE*_RV oligonucleotides and pIJ10257 plasmid (7) as a template. The oligonucleotides contain overlapping fragments and restriction sites for *HindIII* and *XmaI* enzymes to ligate into pIJ10770-*halotag* plasmid using the SLIC method, yielding pIJ10770-*ermE_halotag*. Next, the *cobB1* gene was amplified using *cobB1*_pSS_FW and *cobB1*_pSS_RV oligonucleotides that included the *histag-*encoding sequence on the 5’ of the forward primer. The product (*his-cobB1*) was subsequently purified from agarose gel and ligated using the SLIC method with the gel-purified, *XhoI*/*NdeI*-digested pIJ10770-*ermE_halotag* plasmid, replacing *halotag* gene, and yielding pIJ10770-*ermE*_*his-cobB1* vector. The SLIC reaction mixture was used to transform chemically competent *E. coli* DH5α cells. The transformants were selected on LB plates supplemented with hygromycin, and obtained pIJ10770-*ermE*_*his-cobB1* plasmid was verified by DNA sequencing using pSS_seq_RV oligonucleotide and subsequently transferred, as described earlier, by conjugation to *S. venezuelae* wild-type and JD01 strains, yielding JD04 (*his*-*cobB1*↑) and JD11 (Δ*cobB1*+*his-cobB1*) strains, respectively. The obtained exconjugants were selected and restreaked on MYM agar plates supplemented with hygromycin and verified by colony PCR using pSS_seq_FW and pSS_seq_RV oligonucleotides.

**His-CobB1 protein overproduction and purification**

To construct the vector for overproduction of N-terminally His-tagged CobB1 protein (His-CobB1), the *cobB1* gene (*vnz_03080*) was PCR-amplified from *S. venezuelae* chromosomal DNA using *cobB1*_FW and *cobB1*_RV oligonucleotides. The amplified gene was subsequently ligated using the SLIC method with a pET-28a(+) vector digested previously with *BamHI* and *NdeI* restriction enzymes, yielding the pET-28a(+)-*hiscobB1* vector (confirmed by plasmid sequencing using T7 oligonucleotide). The SLIC reaction mixture was used to transform chemically competent *E. coli* DH5α cells. The obtained transformants were selected using kanamycin. The plasmid DNA isolated from selected transformants was verified with DNA sequencing, and subsequently used to transform chemically competent *E. coli* BL21 (DE3) ArcticExpress cells.

For protein His-CobB1 overproduction, the *E. coli* BL21 (DE3) ArcticExpress pET-28a(+)-his*cobB1* cells were cultured overnight at 37°C with shaking (180 rpm) in 50 mL LB medium supplemented with kanamycin and gentamicin. The next day, 16 mL of the overnight culture was transferred into 800 mL of fresh LB medium and incubated at 30°C with shaking (180 rpm) to reach an optical density of 0.5. Then, the culture was cooled at 4°C for 30 minutes, and His-CobB1 protein overproduction was induced by the addition of isopropyl-β-D-thiogalactopyranoside (IPTG) to the final concentration of 0.1 mM. The culture was continued overnight at 16°C with shaking (180 rpm). The next day, the cells were collected by centrifugation (4000 g, 10 minutes, 4°C), resuspended in 45 mL of the lysis buffer (50 mM Na_2_HPO_4_ pH 8.0, 300 mM NaCl, 20 mM imidazole, 0.5% Triton X-100), and disrupted by sonication. The cell lysate was clarified by centrifugation (25000 g, 20 minutes, 4°C) followed by supernatant filtration through a 0.45 µm pore filter. Subsequently, the cell lysate was incubated overnight at 4°C with shaking with 1 mL of the Ni-NTA Agarose chromatography resin (Qiagen, Netherlands) equilibrated with the lysis buffer. Then, the resin was transferred onto an empty gravity flow column (Thermo Fisher Scientific, US). The settled resin was washed with 50 mL of the wash buffer (50 mM Na_2_HPO_4_ pH 8.0, 500 mM NaCl, 50 mM imidazole, 0.5% Triton X-100). The bound proteins were eluted with the elution buffer (50 mM Na_2_HPO_4_ pH 8.0, 300 mM NaCl, 500 mM imidazole, 0.5% Triton X-100). The elution fractions containing His-CobB1 recombinant protein were combined and desalted using a PD-10 column (GE Healthcare, US) equilibrated earlier with the storage buffer (50 mM Na_2_HPO_4_ pH 8.0, 300 mM NaCl, 10% glycerol). The fractions collected during purification were analyzed using standard Laemmli acrylamide gel electrophoresis (SDS-PAGE) followed by InstantBlue^®^Coomassie Protein Stain (Abcam, UK) staining (CBB) and Western blotting, using an anti-HisTag antibody (see the Western blotting description). The purified His-CobB1 protein was stored at -20°C.

**His-HupS protein overproduction and purification**

To construct the vector for overproduction of the N-terminally His-tagged HupS protein (His-HupS) the *hupS* gene (*vnz_25950*) was PCR-amplified from chromosomal DNA using *hupS*_SLIC_FW and *hupS*_SLIC_RV oligonucleotides. Next, the PCR product was ligated using the SLIC method with the pET-28a(+) plasmid previously digested with *BamHI* and *NdeI* restriction enzymes, yielding the pET-28a(+)-*hishupS* vector (confirmed by plasmid sequencing using T7 oligonucleotide). The obtained pET28a-*hupS* expression vector was used subsequently to transform the chemically competent *E. coli* BL21 (DE3) pLysS cells.

For protein overproduction, the *E. coli* BL21 (DE3) pLysS pET-28a(+)-*hishupS* cells were cultivated overnight at 37°C with shaking (180 rpm) in 50 mL LB medium supplemented with kanamycin and chloramphenicol. The next day, 16 mL of the overnight culture was transferred into 800 mL liquid LB medium supplemented with kanamycin and chloramphenicol, and incubated at 37°C with shaking (180 rpm) to reach an optical density of 0.5. His-HupS overproduction was induced by the addition of IPTG to a final concentration of 0.5 mM, and the culture was continued for 3 hours at 37°C with shaking (180 rpm). The cells were collected by centrifugation (4000 g, 10 minutes, 4°C), resuspended in 45 mL of buffer A (50 mM Tris-HCl pH 8.0), and disrupted by sonication. The cell lysate was clarified by centrifugation (25000 g, 20 minutes, 4°C) followed by its filtration throughout a 0.45 µm pore filter. The purification of the recombinant His-HupS protein was performed using a fast protein liquid chromatography system (GE ÄKTA FPLC^TM^). In the first step, the cell lysate was loaded onto a 1 mL HiTrap^TM^ SP FF ion exchange chromatography column (Cytiva, US) equilibrated previously with buffer A. Then, the column was washed with 15 mL of buffer A. The bound proteins were eluted with buffer B (50 mM Tris-HCl pH 8.0, 2.5 M NaCl) in a three-step NaCl gradient: 250 (I), 500 (II), and 800 (III) mM NaCl. The fractions I-III were analyzed using standard Laemmli acrylamide gel electrophoresis (SDS-PAGE) followed by InstantBlue^®^Coomassie Protein Stain (Abcam, UK) staining (CBB) or Western blotting using anti-HisTag antibody (see the Western blotting description). The fractions containing recombinant His-HupS protein were pooled together and desalted using a PD-10 column (GE Healthcare, US) equilibrated earlier with buffer C (50 mM Tris-HCl pH 8.0, 300 mM NaCl). The purified His-HupS protein was stored subsequently at 4°C.

**Table S1. *Escherichia coli* strains used in the study.**

| **Strain name** | **Relevant genotype and characteristics** | **Source** |
| --- | --- | --- |
| DH5α | *F-,* Φ*80dlacZ*Δ*M15, recA1, endA1*, *gyrA96, thi-E1, hsdR17, (rk-,mk+), supE44, relA1, deoR,* Δ*(lacZYA-argF)U169* | Laboratory stock |
| ET12567/pUZ8002 | *dam, dcm, hsdS,*CmR*,* TetR*,* pUZ8002*: tra,* KanR*, RP4 23;* | Laboratory stock (10) |
| BL21 (DE3) pLysS | F−, ompT, hsdSB (rB−, mB−), dcm, *gal*, λ(DE3), pLysS, CmR | Promega (US) |
| BL21 (DE3) Arctic Express | F−, ompT, hsdS (r−, m−), dcm^+^ TetR, gal λ(DE3), endA, Hte (cpn10 BB cpn60 GentR) | Agilent Technologies Inc. (US) |

**Table S2. *Streptomyces venezuelae* strains used in the study.**

| **Short name (strain name)** | **Relevant genotype and characteristics** | **Source** |
| --- | --- | --- |
| **Wild-type** (WT) | Wild type *S. venezuelae*  NRRL B-65442 (number in NRRL culture collection *,* genome *NZ_CP018074.1*) | Kind gift from prof. Mark Buttner, John Innes Centre, Norwich, UK |
| ***ΔcobB1*** (JD01) | WT *ΔcobB1* | This study |
| ***his-cobB1*↑** (JD04) | WT *attBφBT1*:: pIJ10770-*ermE-hiscobB1* (Hyg^R^) | This study |
| ***hupS-halotag*** (AZ01) | WT *attBφBT1*:: pIJ10770-*hupS-halotag* (Hyg^R^) | This study |
| ***ΔcobB1+hupS-halotag*** (JD14) | *ΔcobB1, attBφBT1*:: pIJ10770-*hupS-halotag* (Hyg^R^) | This study |
| ***ΔcobB1*+*his-cobB1*** (JD11) | *ΔcobB1, attBφBT1*:: pIJ10770-*ermE-hiscobB1* (Hyg^R^) | This study |
| ***hupS-flag*** (TM015) | *ΔhupS::apra, attBφBT1*:: pIJ10770-*hupS-FLAG* (Apr^R^, Hyg^R^) | (10) |
| ***ΔhupS*** (AKO200) | *ΔhupS::apra* (Apr^R^) | (10) |
| **Δ*cobB2*** (JD07) | WT *ΔcobB2* | This study |
| **Δ*cobB1* Δ*cobB2*** (JD08) | WT *ΔcobB1 ΔcobB2* | This study |

**Supplementary Table 3. Oligonucleotides used in the study.**

| **Category** | **Primer** | **Sequence** | **Description** |
| --- | --- | --- | --- |
| **Construction of pCRISPomyces -2_*cobB1*-MH plasmid and strains selection** | oligo_*cobB1*_FW | ACGCTCCCAGAACCAGCAGCGAAT | Preparation of *cobB1*_sgRNA |
|  | oligo_*cobB1*_RV | AAACATTCGCTGCTGGTTCTGGGA |  |
|  | Seq_oligo_fwd | GTGTGAAACTTCTGTGAATG | Verification of *cobB1*_sgRNA and *cobB2*_sgRNA by DNA sequencing |
|  | *cobB1*_FR1_FW | TCGGTTGCCGCCGGGCGTTTTTTA  TCTAGAGAGCGGTGGGTGAGAGT  CAAG | Amplification of 1-kb DNA fragments located upstream (FR1) or downstream (FR2) the *cobB1* gene |
|  | *cobB1*_FR1_RV | CTTCCTCGTGGTCGTCCTCGGTGG  GGGTCCAGC |  |
|  | *cobB1*_FR2_FW | CCCACCGAGGACGACCACGAG  GAAGGCGAGGGC |  |
|  | *cobB1*_FR2_RV | GCGGCCTTTTTACGGTTCCTGGCC  TCTAGAGCTCACCGCCGTACGC |  |
|  | Del_*cobB1*_FW | CCGCACCTGCACGGAAACGAAGGAAACGTG | Selection of *cobB1* deletion strains |
|  | Del_*cobB1*_RV | ACTGCTGGGCCGGGCACGCTCCGACCTCCG |  |
| **Construction of pCRISPomyces -2_*cobB2*-MH plasmid and strains selection** | oligo_*cobB2*_FW | ACGCTGTGCACGGCCTGCCACGCCCGG | Preparation of *cobB1*_sgRNA |
|  | oligo_*cobB2*_RV | AAACCCGGGCGTGGCAGG  CCGTGCACA |  |
|  | *cobB2*_FR1_FW | TCGGTTGCCGCCGGGCGTTTTTTATCTAGAGGGACGCGACGCTGACCAG | Amplification of 1-kb DNA fragments located upstream (FR1) or downstream (FR2) the *cobB2* gene |
|  | *cobB2*_FR1_RV | CGACCATCGTCATCCGGAGTCCGTCGAGATACCG |  |
|  | *cobB2*_FR2_FW | GACGGACTCCGGATGACGATGGTCGGCTCCGCAAGACCGG |  |
|  | *cobB2*_FR2_RV | GCGGCCTTTTTACGGTTCCTGGCCTCTAGAGAGGGCGGCCTGACCC |  |
|  | Del_*cobB2*_FW | AGGACCGTCCGGGACACGATCGCGCTGCACGAGGC | Selection of *cobB2* deletion strains |
|  | Del_*cobB2*_RV | TCACCGCGCACGGCACCCCGGTCGACGA |  |
| **Construction of pIJ10770 plasmid derivatives and strains selection** | SLIC_*ermE*_FW | AGGGGATGATAAGTTTATCAGGTACCAGCCCGACCC | Amplification of *erm*E promoter |
|  | SLIC_*ermE*_RV | TTTCGGATCCATCGTTATTCATATGGGGCCTCCTGTTCTAGAC |  |
|  | *cobB1*_pSS_FW | TCTAGAACAGGAGGCCCCAATGGGCAGCAGCCATCATCATCATCATCACATGCGTATGCGCCCCACCCTG | Amplification of N-terminally His-tagged *cobB1* gene |
|  | *cobB1*_pSS_RV | TCACTGGTACCTTAATTAACTCGAGTCACGGGGCGACCTCGCCGCCCTCGCCTTCC |  |
|  | pss170-linker-HT(C)_fw | ACGTCCTAGGAATAACGATGGATCCGAAATCGGTACTGG | Amplification of *halotag* sequence |
|  | pss170-HT(C)_rv | ACGTCTCGAGTTAACCGGAAATCTCCAGAGTAGACAGCC |  |
|  | *hupS*-tomcherry-2-FW | AGCTCATATGACCGGTTGATAAGGACCTCGACGAGGGC | Amplification of *hupS* gene with its native promoter |
|  | *hupS*-toHT_RV | ACGTCCTAGGCTTCTTGGCGGCGGTCTTCTTGGCGGTC |  |
|  | *hupS_to_mcherry_*fw | AGCTCATATGACCGGTTGATGAAGGACCTCGACGAGGGC | Amplification of *hupS* gene with its native promoter |
|  | *hupS_to_mcherry_*rv | GTCACTCGAGCTTCTTGGCGGCGGTCTTCTTGGCGGT |  |
|  | pSS_seq_FW | AGGATCTTCACCTAGATCCTTTTGGT | Selection of strains carrying pIJ10770 plasmid derivatives |
|  | pSS_seq_RV | GCCAGTGGTATTTATGTCAACACCGC |  |
| **Construction of plasmids for recombinant proteins overproduction** | *hupS*_SLIC_FW | ACGGAGCTCGAATTCGGATCCCTACTTCTTGGCGGCGGTCT | Amplification of *hupS* gene |
|  | *hupS*_SLIC_RV | GTGCCGCGCGGCAGCCATATGGTGAACAAGGCGCAGCTCGT |  |
|  | T7 | TAATACGACTCACTATAGGG | Verification of pET28a(+) plasmid derivatives |
|  | *cobB1*_FW | CATCATCATCATCATCACAGCAGCGGCCTGGTGCCGCGCGGCAGCCATATGATGCGTATGCGCCCCACCCTGAGCTG | Amplification of *cobB1* gene |
|  | *cobB1*_RV | TGCGGCCGCAAGCTTGTCGACGGAGCTCGAATTCGGATCCTCACGGGGCGACCTCGCC |  |
| **Bio-layer interferometry assay** | BLI_*parS*300_FW_Btn | [Btn]GGTAGGTTATCCACGTGTTACTC | Amplification of 5’ biotinylated or 5’ and 3’ biotinylated dsDNA |
|  | BLI_*parS*300_RV_Btn | [Btn]GAACCAGTGAGGCCTGGTCTTC |  |
|  | BLI_*parS*300_RV | GAACCAGTGAGGCCTGGTCTTC |  |
|  | Btn-*parS*-oligo | [Btn]CTCACGCATGCCGGAGTGTCGCGGCAGTTCGGCATCAGCGGCTGTGCATCGTGTTTCACGTGAAACGTCGCTCACTGCTGCACGGCATCATCAGCCGCGGCCGCGCCGCGGCCGACC | Oligonucleotide used in BLI assay (ssDNA) |
| **RT-qPCR** | RT_*cobB1*_FW | GCGTGCTGCCCTGCAT | Relative RT-qPCR analyses of transcript levels |
|  | RT_*cobB1*_RV | GAAGACCACATCCGGCTTGA |  |
|  | RT_*hupS*_FW | GTGGAAGCGATTGCCGACAAGA |  |
|  | RT_*hupS*_RV | CCGAAACCCGTCACAGAGA |  |
|  | RT_*hrdB*_FW | GCCGAGTCCGAGTCTGTGA |  |
|  | RT_*hrdB*_RV | CTGGGTTGGCGGAATCTGGT |  |
| **pUC19A7 plasmid construction** | H24*parA*SmaRV | GCCCAGTACCAGCATCGCTTGG | Amplification of 500 bp fragment of *S. coelicolor* *parAB* operon upstream region |
|  | *parA*pset | CCGGATCCACACAAGCTGCCCTGC |  |

**Table S4. Plasmids used in the study**

| **Plasmid** | **Relevant genotype or characteristics** | **Source** |
| --- | --- | --- |
| **pCRISPomyces-2** | *oriT*, *rep*pSG5(ts), *ori*ColE1, *sSpcas9*, synthetic guide RNA cassette (Apr^R^) | (1) |
| **pCRISPomyces-2_*cobB1*** | pCRISPomyces-2 derivative containing *cobB1_*sgRNA (Apr^R^) | This study |
| **pCRISPomyces-2_*cobB1*-MH** | pCRISPomyces-2_*cobB1* derivative containing 2-kb (FR1+FR2) editing template (Apr^R^) | This study |
| **pCRISPomyces-2_*cobB2*** | pCRISPomyces-2 derivative containing *cobB2_*sgRNA (Apr^R^) | This study |
| **pCRISPomyces-2_*cobB2*-MH** | pCRISPomyces-2_*cobB2* derivative containing 2-kb (FR1+FR2) editing template (Apr^R^) | This study |
| **pIJ10257** | pSET152 plasmid derivative | (7) |
| **pIJ10770** | pMS82^5^ derivative, integrating vector (*attBΦBT1*) (Hyg^R^) | (6) |
| **pIJ10770-*ermE*** | pIJ10770 derivative (*attBΦBT1*), containing the constitutive *erm*Ep promoter (HygR) | This study |
| **pIJ10770-*halotag*** | pIJ10770 derivative (*attBΦBT1*) containing *halotag* sequence (Hyg^R^) | This study |
| **pIJ10770-*ermE*_*halotag*** | pIJ10770 derivative (*attBΦBT1*) containing *halotag* sequence (Hyg^R^)  and the constitutive *ermE*p promoter | This study |
| **pIJ10770-*ermE_his-cobB1*** | pIJ10770 derivative (*attBΦBT1*), his- *cobB1* gene sequence under the control of the constitutive *erm*Ep promoter (Hyg^R^) | This study |
| **pIJ10770-*hupS-mcherry*** | pIJ10770 derivative (*attBΦBT1*), *hupS-mcherry* gene sequence under the control of the native *hupSp* promoter (Hyg^R^) | This study |
| **pIJ10770-*hupS-halotag*** | pIJ10770 derivative (*attBΦBT1*),  *hupS-halotag* gene sequence under the control of the native *hupSp* promoter (Hyg^R^) | This study |
| **PFC 20K HaloTag T7 SP6 Flexi Vector** | - | (5) |
| **pGEM-T-Easy** | Amp^R^ | Promega |
| **pGEM-*hupS*** | pGEM-T-Easy derivative containing *hupS* gene sequence (Amp^R^) | This study |
| **pET-28a(+)** | Plasmid for recombinant protein overproduction in *E. coli* (Kan^R^) | Novagen |
| **pET-28a(+)-*hishupS*** | pET28a(+) derivative, containing *his*-*hupS* gene sequence (Kan^R^) | This study |
| **pET-28a(+)-*hiscobB1*** | pET28a(+) derivative, containing *his*- *cobB1* gene sequence (Kan^R^) | This study |
| **pUC19A7** | pUC19 high copy plasmid derivative containing 500 bp fragment of *S. coelicolor* *parAB* operon upstream region | This study |

**Literature**

1. Cobb,R.E., Wang,Y. and Zhao,H. (2015) High-efficiency multiplex genome editing of Streptomyces species using an engineered CRISPR/Cas system. *ACS Synth Biol*, **4**, 723–728.

2. Engler,C., Kandzia,R. and Marillonnet,S. (2008) A one pot, one step, precision cloning method with high throughput capability. *PLoS One*, **3**, e3647.

3. Holland,R.A. (2023) A Sequence- and Ligation-Independent Cloning (SLIC) Procedure for the Insertion of Genes into a Plasmid Vector. *Methods Mol Biol*, **2633**, 25–32.

4. Kieser,T., Bibb,M.J., Buttner,M.J., Chater,K.F. and Hopwood,D.A. (2000) Practical Streptomyces Genetics The John Innes Foundation, John Innes Centre, Norwich, England.

5. Banaz,N., Mäkelä,J. and Uphoff,S. (2019) Choosing the right label for single-molecule tracking in live bacteria: side-by-side comparison of photoactivatable fluorescent protein and Halo tag dyes. *J Phys D Appl Phys*, **52**, 064002.

6. Schlimpert,S., Wasserstrom,S., Chandra,G., Bibb,M.J., Findlay,K.C., Flärdh,K. and Buttner,M.J. (2017) Two dynamin-like proteins stabilize FtsZ rings during Streptomyces sporulation. *Proc Natl Acad Sci U S A*, **114**, E6176–E6183.

7. Hong,H.-J., Hutchings,M.I., Hill,L.M. and Buttner,M.J. (2005) The role of the novel Fem protein VanK in vancomycin resistance in Streptomyces coelicolor. *J Biol Chem*, **280**, 13055–13061.

8. Stuttard,C. and Dwyer,M. (1981) A new temperate phage of streptomyces venezuelae: morphology, DNA molecular weight and host range of SV2. *Can J Microbiol*, **27**, 496–499.

9. Practical Streptomyces Genetics.

10. Gust,B., Chandra,G., Jakimowicz,D., Yuqing,T., Bruton,C.J. and Chater,K.F. (2004) Lambda red-mediated genetic manipulation of antibiotic-producing Streptomyces. *Adv Appl Microbiol*, **54**, 107–128.
