# Supplementary material for: The activity of CobB1 protein deacetylase contributes to nucleoid compaction in Streptomyces venezuelae spores by increasing HupS affinity for DNA
Source: Nucleic Acids Res. 2024 May 23;52(12):7112–28. doi: 10.1093/nar/gkae418 (PMC11229371; doi:10.1093/nar/gkae418)
Supplement: gkae418_Supplemental_Files [file gkae418_supplemental_files.zip › Supplementary_figures_REVISED2.pdf]

A

```
                                  HU-domain (1-89)
Sv_HupS   VNKAQLVEAIADKMGG-RQQAAEAVDHVLDAIVRAVVGGDRVSVTGFGSFEKVDRPARYA      59
Ec_HUα    MNKTQLIDVIAEKAELSKTQAKAALESTLAAITESLKEGDAVQLVGFGTFKVNHRAERTG      60
          :**:**::.**:*    : **  *:: .* **..::  ** *.:.***:*:  .*  * .
               HU-domain (1-89)                   LR-domain (90-234)
Sv_HupS   RNPQTGERVRVKKTSVPRFRAGQGFKDLVSGSKKLPKGGEVSVKKAPKGSLTGGASATVK      119
Ec_HUα    RNPQTGKEIKIAAANVPAFVSGKALKDAVK------------------------------      90
          ******:.:::  :.** * :*:.:** *.
                                 LR-domain (90-234)
Sv_HupS   KAAAKKATTAKKAAAKKATPAKKTATAAAKKATPAKKTTTAAAKKTSAAAKKTTTAAAKK      179
Ec_HUα    ------------------------------------------------------------      90

                                 LR-domain (90-234)
Sv_HupS   TSAATKKTTTAAAKKATKATAKKTAPAAKKATATKAPAKKTTARKTTAKKTAAKK      234
Ec_HUα    -------------------------------------------------------      90
```

B

```
Sc_CobB1   MRMRPTLSWTPGADLPPGTTDLAPVADALRAGGVLVLSGAGISTESGIPDYRGEGGSLSR     60
Sv_CobB1   --MRPTLSWTPTEDLPPGTTSLAPVTDALRAGGVLVLTGAGISTESGIPDYRGAGGSLSR     58
             *********  *******.****:***********:*************** ******

Sc_CobB1   HTPMTYQDFTAHPEARRRYWARSHLGWRTFGRARPNAGHRSVAAFGRHGLLTGVITQNVD     120
Sv_CobB1   HTPMTYQEFTADARARRRYWARSHLGWRTFGRARPNTGHRAVAAFGRHGLLTGVITQNVD     118
           *******:***. .**********************:***:*******************

Sc_CobB1   GLHQAAGSEGVVELHGSLDRVVCLSCGVLSPRRELARRLEEANAGFSPVAAGINPDGDAD     180
Sv_CobB1   GLHQSAGSEGVVELHGSLERVVCLSCGAFSPRRELARRLEEANAGFAPTAAGLNPDGDAD     178
           ****:*************:********.:*****************:*.***:*******

Sc_CobB1   LTDEQVGDFRVVPCAVCGGVLKPDVVFFGENVPPRRVEHCRELVRGASSLLVLGSSLTVM     240
Sv_CobB1   LTDEQVGDFRVLPCIVCGGVLKPDVVFFGEAVPPQRVEHCRELVDQADSLLVLGSSLTVM     238
           ***********:** *************** ***:*********  *.************

Sc_CobB1   SGLRFVRQAAEAGKPVLIVNRDATRGDRLAVTRVALPLGPALTTVADRLGLRVGDAATA*     299
Sv_CobB1   SGLRFVRQAAQAGKPVLIVNRDPTRGDRHAVTRVALPLGAALTTAAARLGIPVDDQTAGR     298
           **********:*********** ***** ********** ****.* ***: *.* ::.

Sc_CobB1   -------------    299
Sv_CobB1   DHEEGEGGEVAP*    310
```

Supplementary Figure S1. The amino acid sequence of *S. venezuelae* HupS and CobB1 proteins. (**A**) The alignment of *S. venezuelae* HupS (*Sv*_HupS) and *E. coli* HUα (*Ec*_Huα) homologues. The localization of the HU domain (1-89 aa) and LR domain (90-234 aa) are marked with black or blue-dotted lines, respectively. (**B**) The alignment of CobB1 homologues from *S. coelicolor* (*Sc*_CobB1) and *S. venezuelae* (*Sv*_CobB1). Both analyses were performed using the Clustal Omega Multiple Software Alignment Tool. An asterisk (*) indicates the position of a fully conserved residue, and a colon (:) or period (.) indicates the conservation of groups of strongly or weakly similar properties.

A

(A) HupS-FLAG pull-down

Peptide MH+ (Da)	RT (min)	IMS Drift (bins)	MH+ Error (ppm)	No. of fragment matches	Matched BY fragments (° indicates a neutral loss fragment)	Fragments RMS Mass Error (ppm)	PLGS score	Peptide start	Peptide end	Sequence (K* indicates AcLys residue)
1893,98	49,2	52	-3,0	12	b3°b4°b8°b11y3y4y5°y6y9y12 y14y16	7,4	8,00	41	57	(R)VSVTGFGSFEK*VDRPAR(Y)
1335,69	45,7	60	-2,3	19	y2y3y3°y4y5y6y6°y7y8y10	9,0	8,35	80	92	(R)AGQGFK*DLVSGSK(K)
1514,85	32,2	46	-0,2	12	b6b7b13y2y3y4y5y6y7y8y9y12	10,0	8,44	104	119	(K)K*APKGSLTGGASATVK(K)
1218,67	32,9	56	-2,5	14	b4b4°b7°y2y3y4y5y6y7y8y9y9° y10y11	5,1	8,35	108	120	(K)GSLTGGASATVK*K(A)

(B) Cell lysate (WT strain)

Peptide MH+ (Da)	RT (min)	IMS Drift (bins)	MH+ Error (ppm)	No. of fragment matches	Matched BY fragments	Fragments RMS Mass Error (ppm)	PLGS score	Peptide start	Peptide end	Sequence
2307,26	59,2	56	-1,1	3	b5y11y12°	5,1	7,66	72	92	(K)KTSVPRFRAGQGFK*DLVSGSK(K)
3106,83	53,4	86	-1,5	2	b15°b21°	0,0	7,47	94	125	(K)LPKGGEVSVKKAPKGSLTGGASATVK*KAAAKK(A)
1761,06	44,5	75	2,6	3	b10b12y10	7,8	7,45	186	202	(K)KTTTAAAK*KATKATAKK(T) or (K)KTTTAAAKK*ATKATAKK(T)

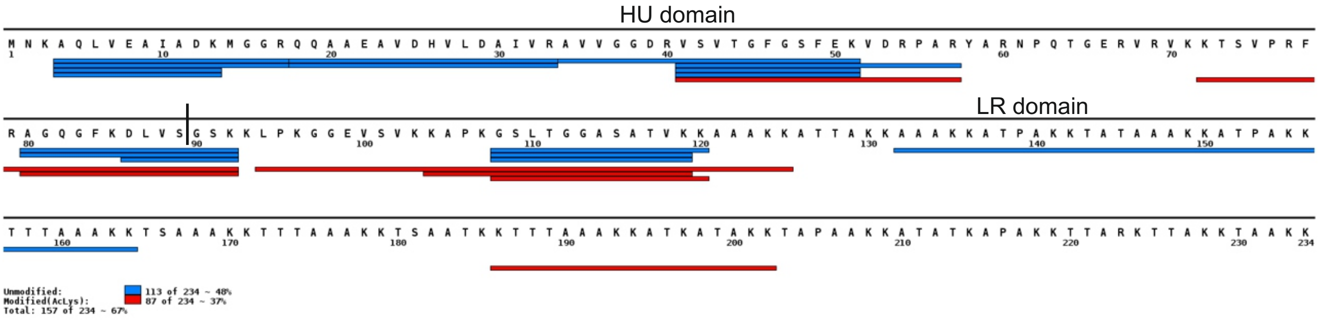

B

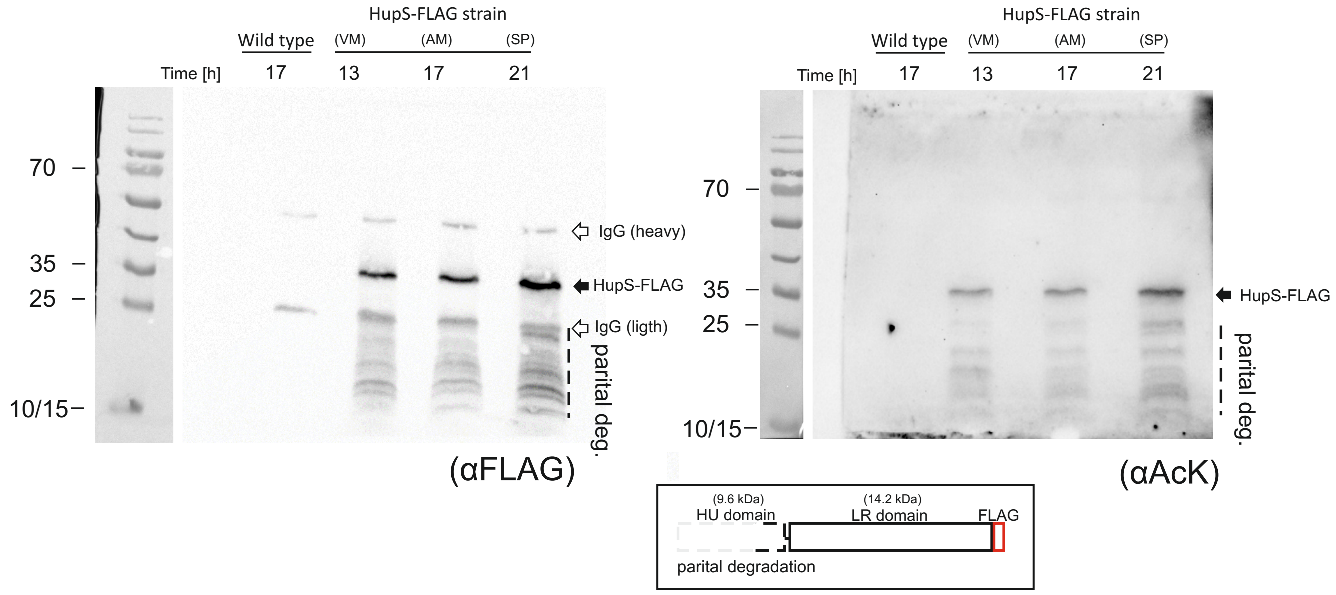

C

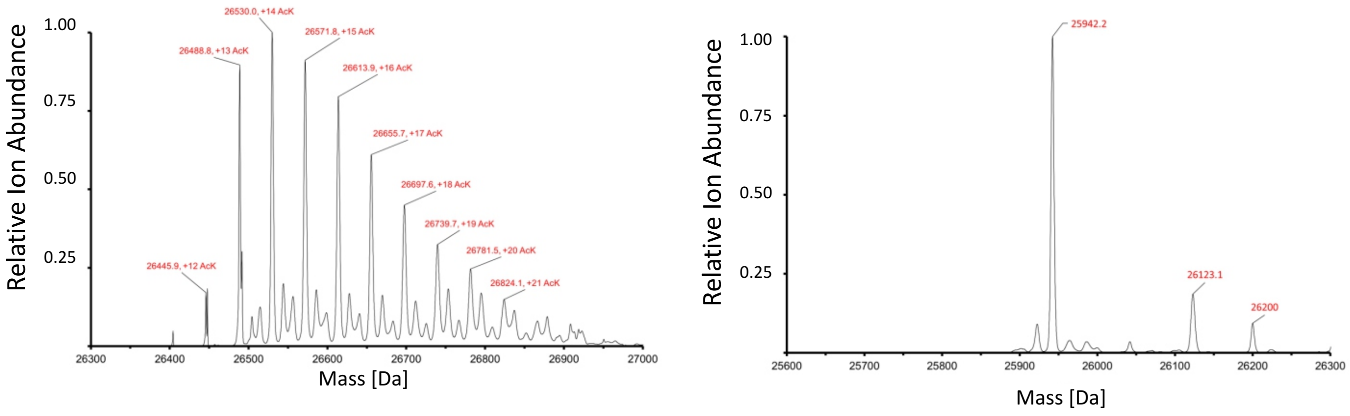

Supplementary Figure S2. Identification of HupS acetylation sites *in vivo* and *in vitro.* (A) Identification of lysine-acetylated peptides of purified HupS-FLAG protein (pulldown experiment, blue panel) or proteomic studies on the cell extract (cell lysate, green panel) obtained from the wild-type *S. venezuelae* strain growing for 21 hours in MYM medium. K* indicates the acetylated lysine residue in the identified peptides. The LC-MS peptide coverage of HupS protein showing the unmodified (blue) and modified (red) peptides is presented below. (**B**) The complete view of the Western blotting with anti-FLAG (αFLAG) and anti-acetyllysine (αAcK) analysis of FLAG pulldown eluent. The *S. venezuelae* cell lysates were obtained at different developmental stages (vegetative (VG) and aerial (AM) mycelium, or spore maturation (SP)) from *S. venezuelae hupS-flag* strain (TM015) growing for 13, 17 and 21 hours in liquid MYM medium. HupS-FLAG is marked with a black arrow. The co-eluted heavy and light chains of bead-immobilized IgG, detected with anti-mouse IgG (αFLAG) but not with anti-rabbit IgG (αAcK) are marked with white arrows. Partially degraded polypeptides co-eluted with intact HupS-FLAG are marked with dashed lines. The scheme of C-terminal HupS-FLAG degradation is shown below. (**C**) LC-MS identification of His-HupS multiple acetylation (left panel) compared with the nonacetylated His-HupS protein (right panel). The molecular weight and the number of acetylated lysine residues are marked in red. The Y-axis shows the ion abundances concerning the highest identified peak (estimated as 1.0).

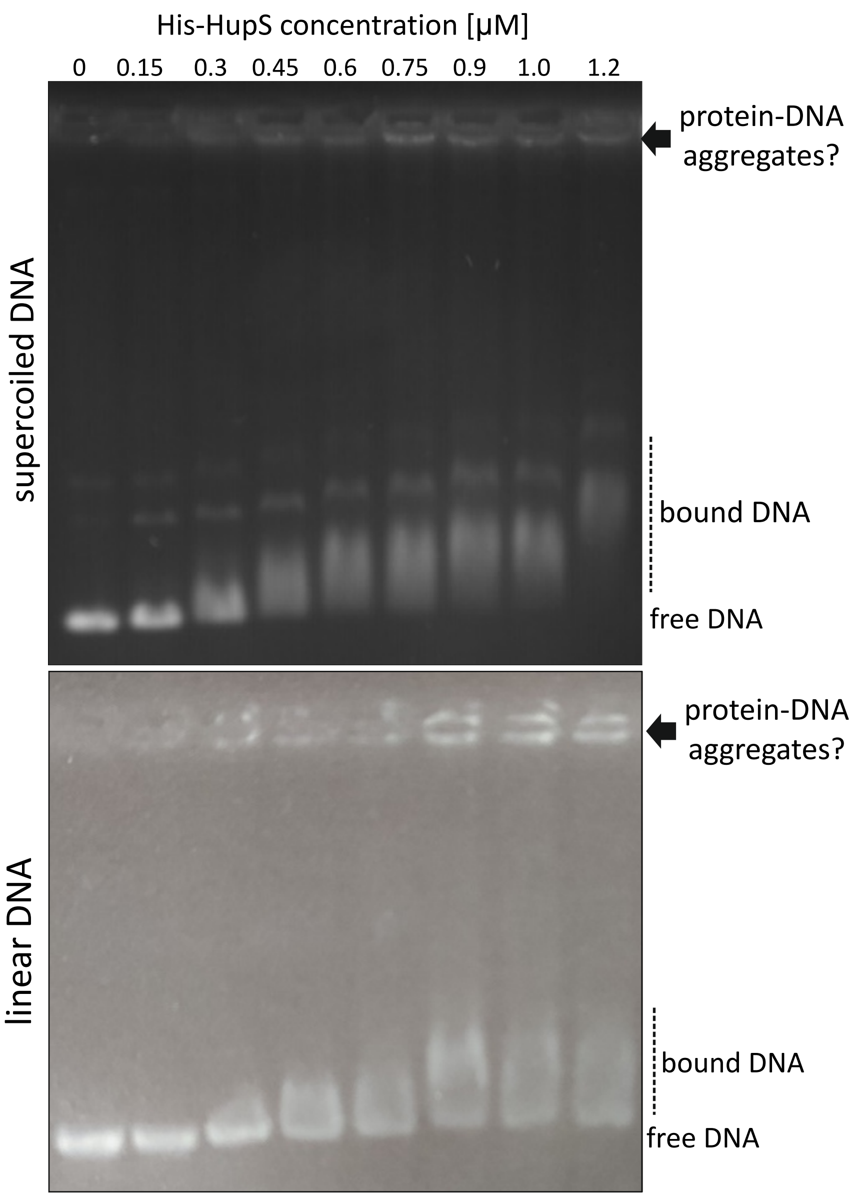

Supplementary Figure S3. Analysis of His-HupS binding *in vitro*. The electrophoretic mobility shift assay was performed in a broad range (0-1.2 µM) of recombinant His-HupS concentrations in the presence of 200 ng of supercoiled (top panel) or linearized (*XbaI*-digested, bottom panel) plasmid DNA. The protein–DNA complexes were resolved overnight in 0.8% agarose gel in TBE buffer followed by ethidium bromide staining. The bound DNA is marked with dotted lines. The black arrows indicate the protein-DNA aggregates.

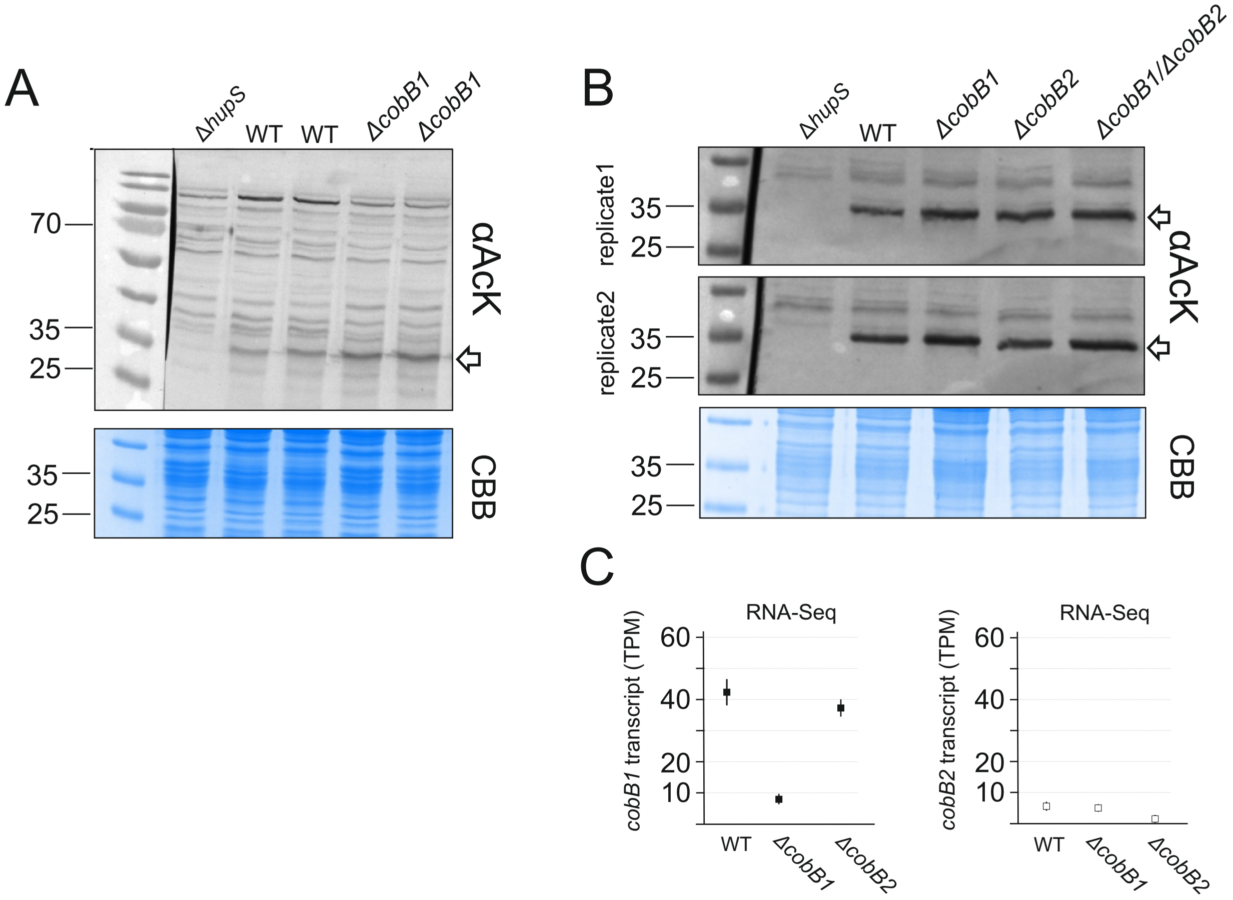

Supplementary Figure S4. Detection of HupS acetylation *in vivo* in the *cobB1* deletion strain. (A) Western blot detection of lysine acetylation (αAcK) in *S. venezuelae* cell lysates obtained from the *ΔhupS* (AKO200), wild type (WT), and *ΔcobB1* (JD01) strains. Replicates no. 2 and 3 of the experiment that is presented in Figure 3B. The band corresponding to HupS is marked with a white arrow. The protein loading control (CBB) is shown below. (**B**) Western blot detection of lysine acetylation (αAcK) in *S. venezuelae* cell lysates obtained from the *ΔhupS* (AKO200), wild type (WT), *ΔcobB1* (JD01), *ΔcobB2* (JD07), and *ΔcobB1/ΔcobB2* (JD08) strains growing for 21 hours in liquid MYM medium. The experiments were performed in two replicates. The band corresponding to HupS is marked with a white arrow. The protein loading control (CBB-stained acrylamide gel) is shown below. (**C**) The levels of *cobB1* and *cobB2* transcripts (transcripts per million, TPM) in the wild-type (WT), *ΔcobB1* (JD01), and *ΔcobB2* (JD07) strains growing for 20 h in liquid MYM medium (spores maturation phase).

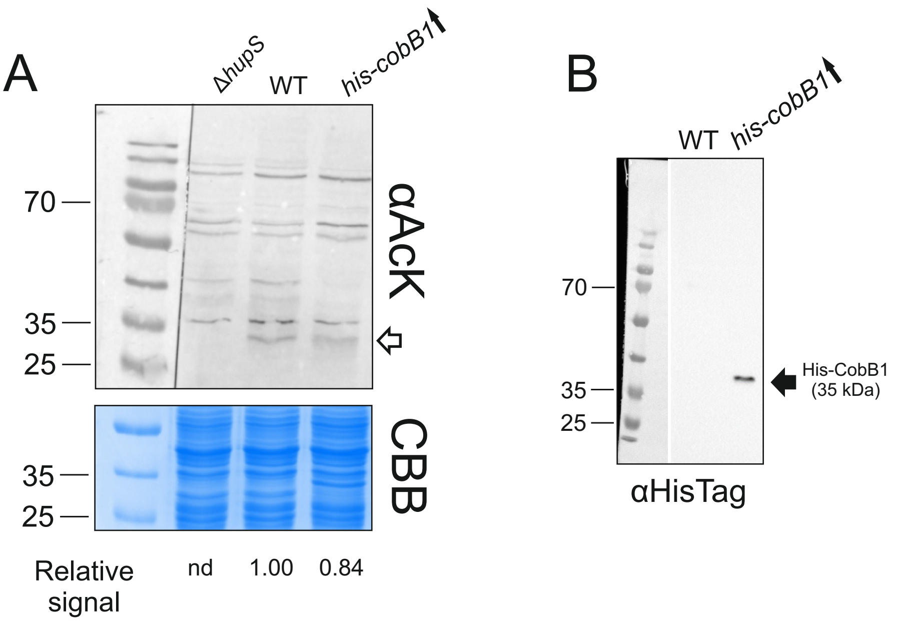

Supplementary Figure S5. Detection of HupS acetylation *in vivo* in the His-CobB1-overproducing strain. (A) Western blot detection of lysine acetylation (αAcK) in *S. venezuelae* cell lysates obtained from the *ΔhupS* deletion (AKO200), wild-type (WT), and CobB1-overproducing (*his-cobB1,* JD04) strains. The band corresponding to HupS is marked with a white arrow. The protein loading control (CBB) is shown below. The relative HupS acetylation signals were quantified and shown below. (**B**) Western blot detection of the His-CobB1 protein with anti-HisTag antibody (αHisTag) in the *S. venezuelae* cell lysates obtained from wild-type (WT) and Cobb1-overproducing (*his-cobB1* ↑ *,* JD04) strains. The band corresponding to His-CobB1 is marked with a black arrow. The protein loading control (CBB) is shown below.

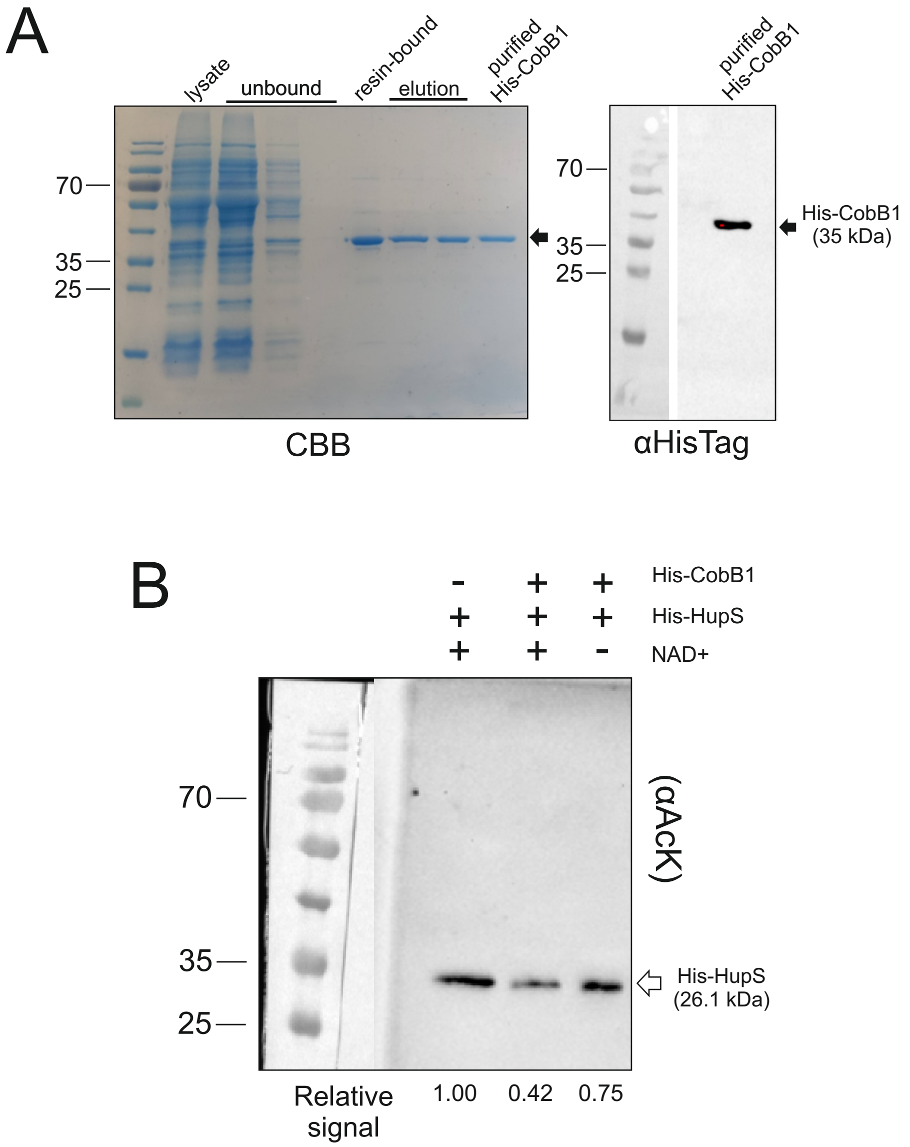

Supplementary Figure S6. His-CobB1 recombinant protein purification and its deacetylase activity *in vitro*. **(A)** Left panel: His-CobB1 (35 kDa) purification steps using nickel affinity. The CBB-stained gel shows particular purification steps, including cell lysate, flow-through fractions (unbound), resin-bound proteins, and imidazole-eluted fractions containing His-CobB1 protein (elution). The last sample shows the purified and imidazole-free His-CobB1 protein. Right panel: Western blot detection of the purified His-CobB1 protein using an anti-HisTag antibody (αHisTag). (**B**) His-CobB1 protein deacetylase activity was detected by Western blotting. The reaction was performed in the presence of acetylated His-HupS protein and in the presence or absence of NAD^+ according to the protocol described in the Materials and Methods. Protein acetylation was detected using an anti-AcK antibody (αAcK). The 25, 35, and 70 kDa bands of the protein molecular weight ladder are marked on the left. The relative HupS acetylation signals were quantified and shown below.

A

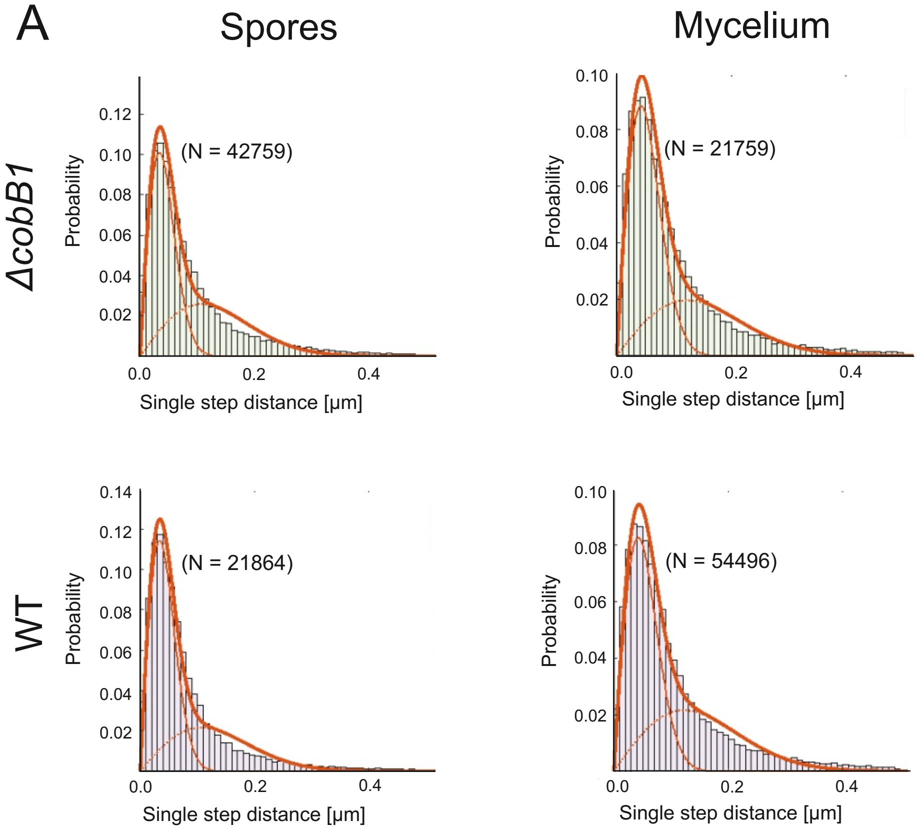

Tracks analyzed

strain	*ΔcobB1*	WT
spores	5754	2801
mycelium	4530	10357

B

Fraction (%) of HupS-HaloTag with low mobility

	WT			*ΔcobB1*			
	Outcome	Exp1	Exp2	Outcome	Exp1	Exp2	Method
mycelium	46.4	46.2	48.5	50.2	49.3	54.4	SQD
mycelium	43.6	42.4	45.6	47.5	46.7	51.1	GMM
spores	66.0	63.4	68.8	58.5	61.6	58.3	SQD
spores	64.9	61.7	67.8	58.2	61.6	57.3	GMM

C

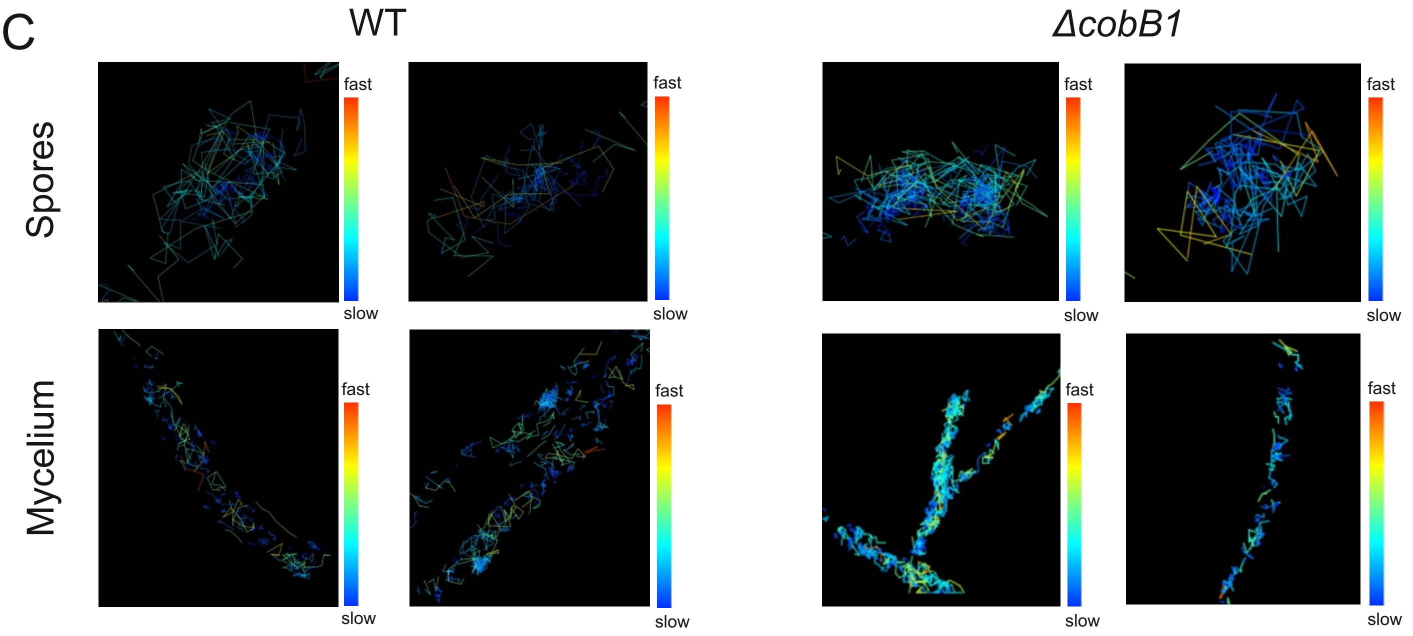

Supplementary Figure S7. Single-particle tracking (SPT) of HupS-HaloTag in the wild-type and *ΔcobB1* genetic backgrounds. (A) Probability distribution of the frame-to-frame displacements for HupS-HaloTag. The single step distances for HupS-HaloTag molecules (N) were quantified in spores and mycelium of the *hupS-halotag* (AZ01, wild-type background) and *ΔcobB1+hupS-halotag* (JD14, *ΔcobB1* background). The fast diffusive (dotted line) and confined (solid line) particle subpopulations were determined by a two-component model. The number of analysed single particle tracks collected for each strain and growth phase is shown below. (**B**) The fraction (%) of HupS-HaloTag proteins with low mobility identified in spores or mycelium in the wild-type (AZ01) or *ΔcobB1* (JD14) genetic background. Single particle tracking (SPT) was recorded in two independent experiments (Exp1, Exp2), and the quantification of low mobility HupS-HaloTag fraction was performed idenpendently using SQD or GMM methods. Then the data from two experiments were combined and reanalyzed (Outcome). (**C**) Examples of SPT tracks recorded for HupS-HaloTag protein in *S. venezuelae* spores and mycelia in the wild-type (AZ01) or Δ*cobB1* (JD14) genetic background. The color pallet indicates slow (blue) and fast (red) track records.

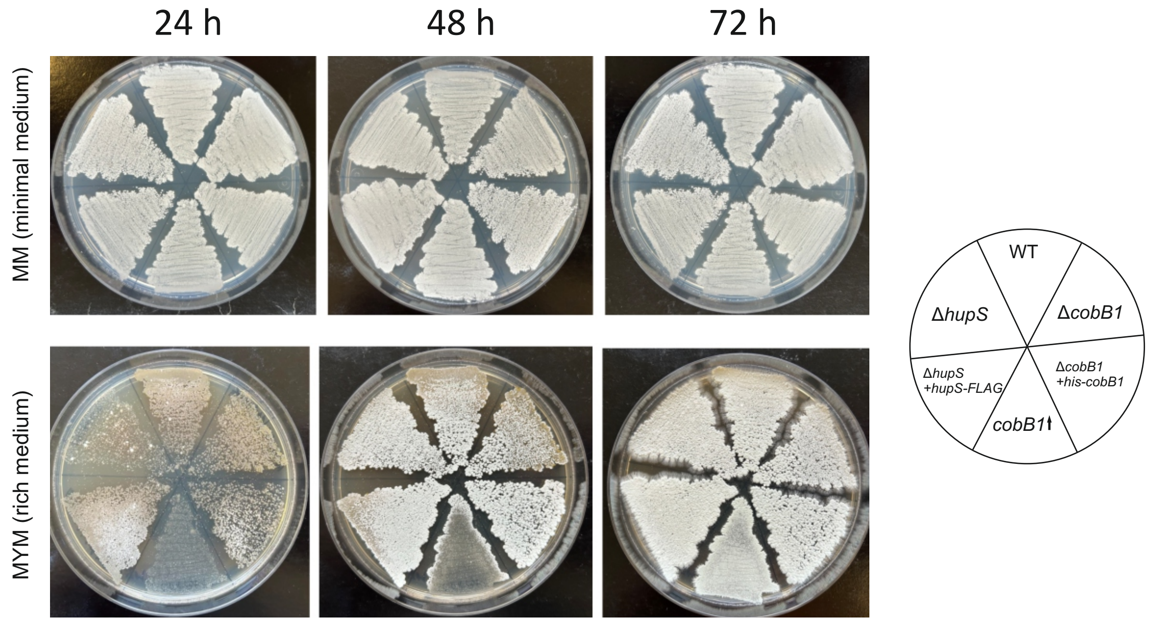

Supplementary Figure S8. The growth of the *S. venezuelae* wild-type (WT), Δ*cobB1* (JD01), Δ*cobB1* complementation (Δ*cobB1*+*his*-*cobB1,* JD11), CobB1 overproduction (*his*-*cobB1* ↑ , JD04), Δ*hupS* (AKO200) on solid MM (minimal medium) or MYM (rich medium) after 24, 48 and 72 hours
